# The Role of Glycemic Index and Glycemic Load in the Development of Real-Time Postprandial Glycemic Response Prediction Models for Patients with Gestational Diabetes

**DOI:** 10.3390/nu12020302

**Published:** 2020-01-23

**Authors:** Evgenii Pustozerov, Aleksandra Tkachuk, Elena Vasukova, Aleksandra Dronova, Ekaterina Shilova, Anna Anopova, Faina Piven, Tatiana Pervunina, Elena Vasilieva, Elena Grineva, Polina Popova

**Affiliations:** 1 Department of Biomedical Engineering, Saint Petersburg State Electrotechnical University, 197341 Saint Petersburg, Russia; 2 Institute of Endocrinology, Almazov National Medical Research Centre, 194156 Saint Petersburg, Russia; aleksandra.tkachuk.1988@mail.ru (A.T.); elenavasukova2@gmail.com (E.V.); aleksandra-dronova@yandex.ru (A.D.); katia.shilova@gmail.com (E.S.); anchylove@mail.ru (A.A.); faina.zernowa@yandex.ru (F.P.); elena-almazlab@yandex.ru (E.V.); grineva_e@mail.ru (E.G.); pvpopova@yandex.ru (P.P.); 3 Department of Gynecology and Endocrinology, The Research Institute of Obstetrics, Gynecology and Reproductology Named after D.O.Ott, 199034 Saint Petersburg, Russia; 4 Institute of Perinatology and Pediatrics, Almazov National Medical Research Centre, 197341 Saint Petersburg, Russia; pervunina_tm@almazovcentre.ru; 5 Department of Internal Diseases and Endocrinology, Pavlov First Saint Petersburg State Medical University, 197022 Saint Petersburg, Russia

**Keywords:** glycemic index, gestational diabetes mellitus, postprandial glycemic response, blood glucose prediction

## Abstract

The incorporation of glycemic index (GI) and glycemic load (GL) is a promising way to improve the accuracy of postprandial glycemic response (PPGR) prediction for personalized treatment of gestational diabetes (GDM). Our aim was to assess the prediction accuracy for PPGR prediction models with and without GI data in women with GDM and healthy pregnant women. The GI values were sourced from University of Sydney’s database and assigned to a food database used in the mobile app DiaCompanion. Weekly continuous glucose monitoring (CGM) data for 124 pregnant women (90 GDM and 34 control) were analyzed together with records of 1489 food intakes. Pearson correlation (*R*) was used to quantify the accuracy of predicted PPGRs from the model relative to those obtained from CGM. The final model for incremental area under glucose curve (iAUC120) prediction chosen by stepwise multiple linear regression had an *R* of 0.705 when GI/GL was included among input variables and an *R* of 0.700 when GI/GL was not included. In linear regression with coefficients acquired using regularization methods, which was tested on the data of new patients, *R* was 0.584 for both models (with and without inclusion of GI/GL). In conclusion, the incorporation of GI and GL only slightly improved the accuracy of PPGR prediction models when used in remote monitoring.

## 1. Introduction

Gestational diabetes mellitus (GDM) has become a common condition during pregnancy, affecting up to 17.8% of pregnancies [1]. GDM is associated with a higher risk of developing serious complications for the mother and the offspring. Short-term pregnancy complications include preeclampsia, macrosomia, birth injury, and increased cesarean delivery rates [1].

Furthermore, apart from promoting the future development of type 2 diabetes (T2D) in the mother [2], GDM is supposed to be an important factor that predisposes an offspring to obesity and type 2 diabetes mellitus (T2D) [3,4]. Given this forecast, maintaining normal blood glucose (BG) levels during pregnancy is critical to curb and reverse the epidemic rise of these conditions [4].

Compliance with diet is the basis of GDM treatment. Food intake is an important determinant of blood glucose levels; consequently, in order to achieve normal glucose levels, it is necessary to make meal choices that induce normal postprandial glycemic responses (PPGRs) [5]. However, the majority of medical organizations do not provide clear recommendations on diet for GDM patients and give only general guidelines. Even if the recommendations are more detailed, these diets description concerns only characteristics of the foods and does not take into account the individual features of patients. However, compelling evidence suggests that glycemic responses to the same food items considerably vary among individuals [6,7]. Apart from the characteristics of the foods consumed, the glycemic responses of individuals associate with multiple person-specific factors [6,7]. In 2015, Zeevi et al. described a machine-learning algorithm for PPGR prediction integrating blood parameters, dietary habits, anthropometrics, physical activity, and gut microbiota measured in healthy individuals in an Israeli cohort [6]. Dietary intervention based on this algorithm resulted in significant improvements in multiple aspects of glucose metabolism, including lower PPGRs and lower fluctuations in blood glucose levels [6]. However, this algorithm has not been studied in pregnant women and in patients with diabetes mellitus, including GDM, to our knowledge, and it requires additional expensive analyses.

Thus, the development of effective methods for selecting the optimal composition of meals for increased PPGR prevention is extremely important for the treatment of patients with GDM.

Personalized BG prediction in healthy subjects and especially patients with diabetes mellitus is an important goal that is pursued by many researchers worldwide [6,8,9,10,11,12]. Neither of them assessed the effectiveness of PPGR prediction in GDM patients. We have developed a recommender system infrastructure that incorporates BG prediction models for GDM patients [13]. It is expected that integration of such models into an interactive mobile app will lead to the creation of personal recommendations for nutrition in real time to prevent hyperglycemia in patients with GDM. Implementation of such an app may improve the effectiveness of treatment and at the same time reduce the burden to healthcare providers through the reduction of time spent for on education concerning diet in GDM.

We have developed algorithms for predicting the following PPGR parameters: BG 60 min after the start of food intake (BG60), peak BG value after food intake (BGMax), area under the glycemic curve 1 (AUC60) and 2 h (AUC120) after the start of the meal and peak BG, and incremental area under the glycemic curve 2 h after food intake (iAUC120) [13]. The accuracy of predicting the AUC60 and AUC120 was adequate, but the model for predicting BG60 was not accurate enough. Further studies are needed to increase the accuracy of the BG prediction at single time points, in particular, 1 h after meals, because this time point was recommended with target values to guide clinical practice [14,15]. These regression models were based on objective and laboratory data, anamnesis, questionnaires, and diaries of pregnant women with GDM and women with normal glucose tolerance. Data derived from diaries included macronutrient and micronutrient content, but did not incorporate glycemic index (GI), because reliable databases describing the GI of different foods are absent in many countries, including Russia. Incorporation of GI and glycemic load (GL) is a promising way to improve the accuracy of PPGR prediction [16,17]. The superiority of dietary GL over carbohydrate content alone for estimating postprandial glycemia has been shown in healthy individuals consuming isoenergetic portions of single foods and mixed meals [16]. Moreover, GI was shown to be the strongest and the most consistent independent predictor of PPGR in a study of free-living people with type 2 diabetes mellitus (T2DM) who kept three-day food records simultaneously with continuous glucose monitoring [17].

The aim of the study was to assign GI to a food database of a Russian institute of nutrition and to assess the prediction accuracy for PPGR prediction models with and without GI data in women with gestational diabetes (GDM) and healthy pregnant women.

## 2. Materials and Methods

### 2.1. Research Methodology

This study involved a subset of women who participated in the GEM-GDM randomized controlled trial (Genetic and Epigenetic Mechanisms of Developing Gestational Diabetes Mellitus and Its Effects on the Fetus) and were recruited between November 2015 and July 2019 in the Almazov National Medical Research Centre (ANMRC). This study was approved by the local ethical committee (Protocol 119), and the participants gave their consent in writing. The protocol of the parent study is reported elsewhere [18]. In brief, the study included pregnant women with GDM and pregnant women with normal glucose tolerance (control group) aged 18–45 years. The women with GDM were randomized into 2 groups according to target glycemic levels: Group 1 (target fasting blood glucose <5.1 mmol/L and <7.0 mmol/L 1-h postprandial) and Group 2 (target fasting blood glucose <5.3 mmol/L and <7.8 mmol/L 1-h postprandial). For the purpose of the study reported here, the women from these two groups were merged and formed the GDM group. The inclusion criteria for the GDM group were as follows: pregnant women with GDM diagnosed according to the Russian national consensus [14] and the recommendations of the International Association of Diabetes and Pregnancy Study Groups (fasting glucose of ≥5.1 mmol/L, and/or ≥10.0 mmol/L after 1 h, and/or ≥8.5 mmol/L after 2 h in oral glucose tolerance test (OGTT) with 75 g of glucose) [19]; a gestational age of <32 weeks at the time of inclusion in the study. Inclusion criteria for the control group were as follows: pregnant women with normal glucose tolerance confirmed by OGTT at 24–31 weeks of gestation. Exclusion criteria were a history of diabetes mellitus or any known medical condition affecting glucose metabolism. Treatment with insulin, although not an exclusion criterion for the parent trial, was exclusionary in the study reported here. Pregnant women were invited to take part in this study if they used our mobile app or our desktop app [20] and provided accurate information concerning their food intake and BG measurements.

The GEM-GDM trial was registered at the ClinicalTrials.gov (Identifier: NCT03610178).

### 2.2. Food Database and Calculation of Glycemic Index

Meal data were recorded with a specially developed app, DiaCompanion [20], with which patients chose food items from a database created by the authors on the basis of reference books of the Russian Academy of Medical Sciences and the US Department of Agriculture (USDA) Food Composition Databases (Release 28). The current database contains mainly foods available in Russia and consists of 2180 records, each of which is classified either as a simple item (*n* = 1245) or a complex dish (*n* = 935).

GI was not initially presented in the database, and it was the task for the current study to match each item in the database with an appropriate GI available in the open glycemic index databases.

Each food recorded in the diaries was assigned a dietary GI according to the method published by Louie et al. [21]. Foods were either assigned (1) a published GI, (2) a GI of 0 for foods with a carbohydrate content below 5 g/100 g (e.g., meats), (3) a published GI of a close match (e.g., peach and apricot), (4) a mean GI of a subgroup of foods (e.g., breads), or (5), for the products without a close match or matching subgroups, a GI value of 0, 50, or a GI value of an appropriate, closest matched item as decided by the research nutritionists. Each GI was assigned in three steps by three independent researchers (endocrinologists): 1st step—initial assignment of GI by a single researcher; 2nd step—the above process was reviewed by another researcher; 3rd step—any discrepancies were finalized in a case-by-case discussion between the two researchers and the senior researcher. To ensure accuracy and appropriateness of the GI values assigned, the whole nutrition database was also reviewed by a senior researcher.

In total there were 175 items assigned directly with the published GI; 436 foods had zero carbohydrates and were assigned zero GI; for 211 items, a published GI of a close match was assigned; for 315 items, the mean GI of a subgroup of foods was assigned; 108 products without a close match or matching subgroups were assigned a GI value of 0, 50, or a GI value of an appropriate, closest matched item as decided by the researchers.

For complex foods, the dietary GI was calculated from the GI values of the food’s ingredients, using recipes available in the in-house database. During the process of matching a particular food with one listed in the tables, the principle consideration was the carbohydrate content of the food. Fat content, protein content, and preparation methods were also considered in the decision-making process in descending order of importance.

The GI values were sourced from the University of Sydney database (www.glycemicindex.com) [22].

After every simple item in the database was manually assigned a GI, GI values for complex dishes were automatically calculated with the following formula:

(1) $$gi=\frac{\sum_{i=1}^{N}gi_{i}carbo_{i}}{\sum_{i=1}^{N}carbo_{i}}$$

where N is the amount of food items in the dish, $gi_{i}$ is the glycemic index for the *i*-th food item, and $carbo_{i}$ is the mass fraction of carbohydrates for the *i*-th food item.

The same strategy was used when calculating the GL for meals containing more than one food item. The appropriate GL for such meals was calculated as

(2) $$gl=\frac{1}{100}\sum_{i=1}^{N}gi_{i}carbo_{i}$$

where N is the amount of food items in the meal, $gi_{i}$ is the glycemic index for the *i*-th food item, and $carbo_{i}$ is the mass fraction of carbohydrates for the *i*-th food item.

In addition to 2178 food items from the database, another 196 complex dishes that were added by patients by means of the app were also manually provided with GI values by the authors. After each food item from the database was matched with the glycemic index, all the collected data on meals for all patients were automatically matched with appropriated GI values, and GI and GL were prepared to be added as inputs for prognostic models.

Altogether in the collected database, there were 611 (25.7%) items assigned a zero GI, 187 (7.9%) items with a GI between 0 and 25, 589 (40.0%) items with a GI between 25 and 50, 949 items with a GI between 50 and 75, and 28 items with a GI higher than 75 (1.6%). The mean GI for the collected database was 38, and the median 44.

**Figure 1:**
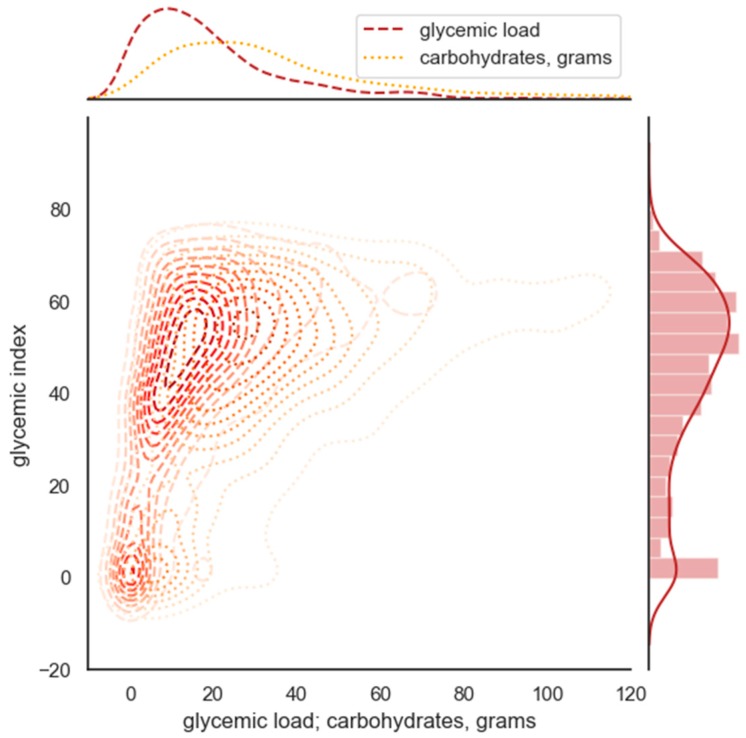
Density plot showing paired distribution of glycemic index (GI) and glycemic load (GL)/carbo in all meals included in the study (*n* = 1489).

Figure 1 shows the pair distribution of GI and GL/carbo in all meals selected for the following model study.

### 2.3. Continuous Glucose Monitoring (CGM) and Meal Data Matching

Continuous glucose monitoring (CGM) was monitored over a period of 4–7 days from 19 to 36 weeks of pregnancy using the iPro2 CGM with Enlite sensors (Medtronic, Minneapolis, MN, USA). For a subset of women (*n* = 24), who were initially monitored before the 33rd week of pregnancy, CGM was repeated in the 36–37th weeks of pregnancy. Second signals for the same patients were treated as data from the same patients, so no data for the same patients appeared twice among the training, validation, and testing sets. Simultaneously, participants tracked records in a paper protocol, in which patients stated the exact time of beginning and completing instances of food intake, together with blood glucose measurements. This paper protocol was initially used because CGM required manual glucose monitoring at least 4 times a day for its calibration, which was performed using the Accu-Check Performa Nano blood glucose meters (Roche Diabetes Care, Indianapolis, IN, USA).

Meal data were collected and exported from the app as Excel spreadsheets (electronic food diaries). Each meal record consisted of meal type, meal time, and a list of food names in the meal with appropriate weights in grams.

One hundred thirty-eight patients had successfully recorded weekly CGM, sent an electronic diary exported from their mobile apps, and returned the paper protocol to their physician. Data on point blood glucose measurements were entered onto the carelink website together with marks on the time of food intake, from which it was downloaded and merged with electronic diaries exported from mobile app by means of the software developed in the current study.

The software for data processing, modeling, and data visualization was written by the authors using the Python 3.7 programming language [23]. The following packages were used for data processing: pandas, numpy, scipy, statistics, math, os, datetime, dateutil, codecs, and sys. For data export xlwt, xlrd, openpyxl, csv, and xlutils packages were utilized. Matplotlib and seaborn were used for visualization and the sklearn package [24] for creating and analyzing blood glucose predictive models.

After CGM and meal data were collected, they were matched using the following strategy. Each food start record in the paper protocol was matched with the nearest record in the electronic food diary. If there were no corresponding meal data in the diary, the records in the protocol were ignored. The meals that had a misreported meal start time or were interfered with other meals were excluded by the following criteria:

- meals with a start time reported significantly later than the actual meal start according to CGM (falling on the peak value in CGM signal), i.e., the BG level at the reported meal start is more than 1.0 mmol/L higher than the BG level 1 h before the meal (*n* = 103).
- meals with a start time reported on the falling edge of the peak, i.e., the BG level at the reported meal start is at least 0.5 mmol/L higher than the BG level in half an hour and at least 0.5 mmol/L lower than half an hour prior (*n* = 32).
- meals with reported prior meals less than 1 h before meal start (*n* = 25).
- meals with subsequent meals less than 1 h after meal start (*n* = 104).

**Figure 2:**
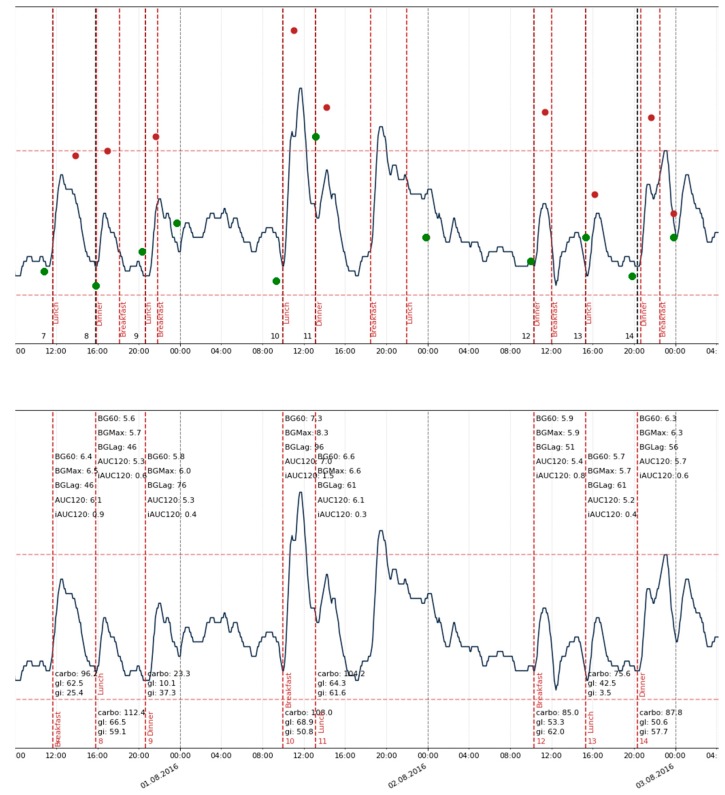
Continuous glucose monitoring (CGM) and food diary matching strategy. On the top: black vertical lines: meal starts written in a paper protocol; red line: meal starts as written in the electronic diary; on the bottom: red lines: meal starts chosen for the final sets; upper marks: postprandial glycemic response (PPGR) features; lower marks: meal features. Green dots represent point estimations of blood glucose (BG) levels made with a glucometer used for sensor calibration, and red points correspond to point estimations of BG 1 h after the meal. It can be seen that meals coming as close as 60 min to each other were ignored, as well as records from the electronic diary, which did not have an exact time specified in the protocol.

An illustration of applying a strategy for CGM and meal data matching is presented in Figure 2. After a selection procedure, there were 1865 records with meal data and corresponding PPGR curves collected for the analysis. Examples of CGM and meal data from patients with meal diaries of excellent, good, and bad quality are presented in Supplementary Material Figure S1.

### 2.4. Data Preprocessing and Filtering

Data from 16 patients (175 records) were excluded due to misreporting (misreporting was detected when there was a significant lack of food data—i.e., all meals consisted only of a single item—or when data were imprecise, i.e., rounded to 100 g for all food reports for more than 5 meal records in the diary).

Acquired data were then filtered in the following steps: 41 records with rarely used dishes, where GI was not defined for a food item, were removed, as were 101 records that have a small PPGR (less than 0.3 mmol/L/h) to meals with more than 40 g of carbohydrates, as proposed by Mendes-Soares et al. [7]. After all filtering procedures, there were 1489 records included in the final evaluation.

### 2.5. Individual Characteristics of Participants

After each postprandial blood glucose curve was matched with meal data from diaries, each record was supplemented with a set of features characterizing each patient. These data included the following:

1. anthropometric and individual parameters (age, weight, body mass index (BMI), gestational age, and systolic and diastolic blood pressure);
2. medical history data (GDM in history, polycystic ovary syndrome, impaired glucose tolerance, family history of diabetes, number of pregnancies, abortions, deliveries, and miscarriages, arterial hypertension, and use of combined oral contraceptive pills before pregnancy).
3. biochemical parameters (fasting, 1-h and 2-h BG levels at OGTT, fasting insulin, HbA1c, fructosamine, leptin, total cholesterol level, very low density and high density lipoproteins, and triglycerides at the time of OGTT);
4. questionnaire data—11 parameters associated with the consumption of certain product groups, 3 parameters related to beverages, and 3 parameters characterizing physical activity. For each listed parameter, the intensity was coded according to an ordinal scale of three levels (0 for low, 1 for medium, and 2 for high). Smoking was marked as “yes” or “no.” All parameters were assessed separately before and during pregnancy. This questionnaire has been previously reported [25,26].

In the dataset characterizing patients, there was a small amount of missing data, which was imputed by the simple single-column imputer assigning a mean feature value for each group of patients (GDM or control) to each missing value. There were no missing data except data characterizing patients.

There were 119 input features chosen for the analysis. Dummy variables were created for every non-ordered categorical input variable via one-hot encoding before they were fed into the model, resulting in 222 input features in total. Dummy-encoded variables were named as “variable_value,” e.g., “fruits_1” and “fruits_2.” The complete list of features used as an input for BG predictive models is shown in Supplementary Material List S2.

### 2.6. Blood Glucose Predictive Models

The characteristics of the PPGRs, which were predicted by the models, were as follows: blood glucose level 60 min after the meal (BG60), peak blood glucose level 3 h after the meal (BGMax), the rise of blood glucose level from the beginning of the meal to the peak value (BGRise), area under the postprandial blood glucose curve 120 min after the meal (AUC120), and incremental area under the blood glucose curve 60 and 120 min after the meal (iAUC60 and iAUC120). iAUC120 was chosen as the primary feature, as it is often referred to as the best characteristic describing PPGR [6].

After all the features and output characteristics were merged in a data frame, two types of models were utilized to evaluation:

- simple stepwise regression, to compare current results with recent publications;
- linear regression with coefficients acquired using regularization methods with cross-validation for feature selection, which was tested on the data of new patients.

For the second type of models, the data were separated into train and test sets in the proportion of 70/30% in a way that none of the data belonging to the same patient were in both sets (grouped). The train data were grouped with the use of a grouped 10-fold cross-validation on the model evaluation stage, where folds were organized in a way where each test set consisted of measures from patients not included in the test set group. The regressors X were normalized before regression by subtracting the mean and dividing by the l2-norm.

Different regularization strategies were tested in both settings, including Lasso, Ridge, Elastic-Net, and LARS lasso. The best result was achieved with LARS lasso regression [27] and orthogonal matching pursuit (OMP) [28] algorithms. *r*^2^ was chosen as a score function of the estimator to evaluate a parameter setting. As both methods tended to overfit the data even in a cross-validation setting, only variables that had a Spearman correlation |*r*| that was >0.1 with the predicted variable were selected for further automatic selection via cross-validation feature selection. Exhaustive Grid Search was used to find the optimal value of alpha parameters (the hyperparameter was chosen with the maximum score on a multiple validation sets). The best model was chosen by the largest *r*^2^ score. It was then evaluated on a 30% test set.

Both OMP and LARS Lasso received relatively similar precision (±0.02 in *r*^2^), thus OMP was chosen as a preferred method for coefficient estimation, as it tended to select a smaller amount of features (more information on OMP and LARS comparison can be found in the work by Hameed [29]). The effect of inclusion of polynomial features was also analyzed in the study.

### 2.7. Statistical Analysis

Data were statistically processed with SPSS 22.0 (IBM Corporation, Armonk, NY, USA) and Python 3.7 (Python Software Foundation, Delaware, DE, USA). Differences in the quantitative characteristics of the groups were assessed with a Student’s *t* test. The chi-square criterion was used to compare the distribution of qualitative characteristics. The differences were considered significant at *p*-value < 0.05. Pearson product moment correlation was used to quantify the accuracy of the predicted PPGRs from the model relative to those obtained from the CGM. It was also used to quantify the correlation between meal content characteristics (gi, gl, carbo, prot, fat, kcal, water, and starch) and PPGRs estimated from the CGM measurements. Features were chosen via 10-fold cross-validation with a coefficient of determination (*r*^2^) as an optimizing parameter. Mean absolute error (MAE) and a coefficient of correlation *R* were estimated for all chosen models.

## 3. Results

### 3.1. Characteristics of Participants

Table 1 contains information on participants included in the study. The women with GDM had higher BMI and higher levels of HbA1c, plasma glucose (PG) during OGTT, and serum triglycerides than the controls. The data are presented as (mean ± standard deviation) pairs.

### 3.2. Correlation Analysis

Table 2. shows the correlation between meal features and PPGR on the complete dataset of meals from all included patients. Interestingly, starch correlated with iAUC120 with almost the same strength as carbo and GL, while GI had only a weak correlation with iAUC120. Of note, GL correlated much more with carbo than with GI (*r* = 0.952 vs. 0.406).

**Figure 3:**
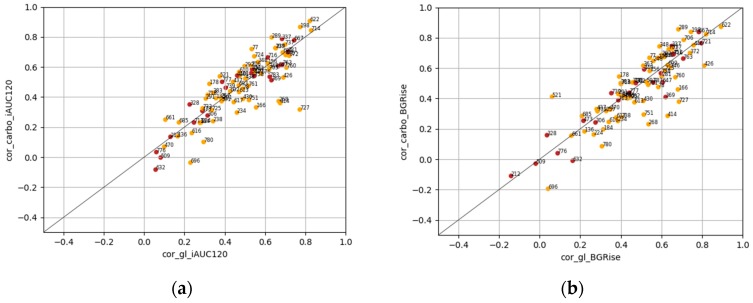
Correlation coefficients between PPGR characteristics (iAUC120 on the left, BGRise on the right) and carbohydrates/glycemic load. The number next to each point depicts a patient’s individual identifier. In figure (**a**): cor_gi_iAUC120: correlation between glycemic load and incremental area under glucose curve 2 h after meal start; cor_carbo_iAUC120: correlation between consumed carbohydrates and incremental area under glucose curve. In figure (**b**): cor_gi_BGRise: correlation between glycemic load and blood glucose rise from meal start to peak value; cor_carbo_BGRise: correlation between consumed carbohydrates and blood glucose rise from meal start to peak value. Orange: GDM group; brown: healthy pregnant participants.

Table 2 shows the averaged correlation coefficients calculated on meal data from all patients. The individual correlation coefficients between the amount of carbohydrates, GL, and PPGR are shown in Figure 3 (patients with 10 or more meal intakes were included). Figure 3 shows high variability in individual relation between carbohydrates/glycemic load and PPGR characteristics (iAUC120 and BGRise) covering the spectrum from a very weak to a high correlation. It also shows the difference in how GL and carbohydrates are correlated with iAUC120 and BGRise in each patient. Only in 48.2% of patients did GL have a larger correlation with iAUC120 compared with the amount of consumed carbohydrates, and for 54.2% patients with BGRise. Examples with individual data from patients with various carbo/GL/PPGR correlations are shown in Supplementary Material Figure S3.

### 3.3. Simple Stepwise Regression

All predictors described in Section 2.5 were entered into a stepwise multiple linear regression model. *R* squared was selected as an optimization parameter. Table 3 shows the list of model scores with selected features on each step. The first step in which carbo was chosen stands for 0.434 of correlation of the model.

The final model chosen by stepwise regression contained 53 input variables and had an *R* of 0.705; *R* squared = 0.497; adjusted *R* squared = 0.482; standard error = 0.471. In case information on GI/GL was not included in the set of input data, the final model included 44 input variables, and its characteristics were the following: *R* = 0.700; *R* squared = 0.490; adjusted *R* squared = 0.475; standard error = 0.474, which shows that information on GI/GL does not play a crucial role in a linear model created on the whole set of meals from all patients.

Table 4 shows the list of coefficients of the first three linear models predicting iAUC120 created with stepwise regression, where information on GI/GL was included in the set of input data. GI/GL was not selected by these three linear models. GI/GL was selected only starting from the 14th step in stepwise regression.

The final models show the limit to which linear models built on the whole set of GDM/control patients can predict PPGR in the current setting on the data presented. The complete set of models predicting iAUC120 with appropriate coefficients achieved with stepwise regression is shown in Supplementary Material Table S4.

The characteristics of appropriate final linear models in which GI/GL was presented as an input variable (with GI/GL) and those not including GI/GL as an input variable (without GI/GL) are shown in Table 5. Overall there was only slight increase in the accuracy of PPGR prediction for each model. For example, for iAUC120 R increased from 0.700 to 0.705 after adding of GI/GL as an input variable (Table 5).

### 3.4. Regularized Regression and Testing on New Patients

To create a model capable of working on data from new patients, appropriate regularized linear models for each PPGR characteristic were created (methodology described in Section 2.6). The prediction quality assessed on the 30% test set with data from new patients for each resulting model (OMP) is shown in Table 6. The prediction of iAUC120 is overall much better than that of BGRise. GI/GL was included as an input variable for each model, but was selected by the regularized regression selection algorithm only for BGMax, AUC120, and iAUC60 (Table 6). Table 7 shows appropriate models, in which polynomial features (e.g., carbo^2^ and carbo × gl) were added.

The addition of polynomial features resulted in a minor improvement for all the models (except one for AUC120 prediction), thus making the models more complicated (with a larger amount of independent variables in each model). The coefficients for regression models with and without added polynomial features are shown in Supplementary Material List S5.

**Figure 4:**
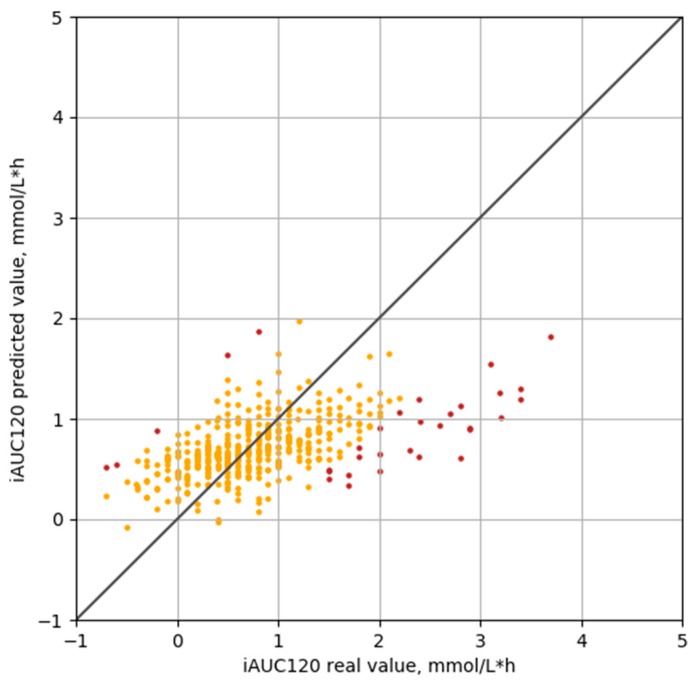
Prediction of iAUC120 on new patients with the regularized regression model (*R* = 0.584). Orange dots depict PPGRs whose errors are equal or below 1.0 mmol/L·h (92.3%), while brown dots depict those whose errors are above 1.0 mmol/L·h (7.7%).

Figure 4 shows the results of iAUC120 prediction on the test set of new patients. There were only a few meals (32 (7.5%) out of 428 meals from 38 patients in the test set) in which predicted iAUC120 differed from real value by more than 1.0 mmol/L·h, mainly (*n* = 28) when the real value was higher than the estimated one.

## 4. Discussion

In this study, we demonstrated that prediction accuracy for PPGR prediction models in women with GDM and healthy pregnant women did not substantially increase after adding GI and GL information to the models utilizing individual participant data and meal characteristics.

Our data contradict the conclusions made by Bao et al. based on their study where GL was the strongest predictor of glycemia after mixed meals, explaining 58% of the observed variation [16]. However, the study by Bao was performed under much stricter conditions: (1) they included a selected group of lean glucose-tolerant individuals, with the likelihood of optimal b cell function, (2) metabolic responses were studied only at breakfast time, (3) repeated testing of a reference food was performed, and (4) specific foodstuff with well-defined GI were used. All these conditions make the results less reproducible in clinical practice. The relations between GL and PPGR seen in this group may not apply to pregnant women, especially those with GDM.

In the study performed in free-living conditions by Fabricatore et al. on adults with DM2 GI accounted only for 10 to 18% of the variance in each glycemic variable, but “GI was the strongest and most consistent independent predictor of glycemic stability and variability” [17]. However, the authors were not able to predict individual PPGR as “the participants did not reliably record the time of food intake; thus, the glycemic response could not accurately be linked to individual intake episodes” [17]. Therefore, the researchers used total daily GI and GL associations with different glycemic variables instead of individual PPGR prediction. This may explain the higher correlation of GI with AUC in comparison to carbohydrate amount with AUC. However, the correlation between GL and AUC in their study was considerably lower than the correlation between GL and iAUC120 in our study (0.29 vs. 0.423). To the best of our knowledge, there are no published studies exploring the utility of using GI/GL for individual PPGR prediction performed in free-living conditions.

In our study, in only 50% of patients did GL have a larger correlation with iAUC120 compared with the amount of consumed carbohydrates. We also observed a high interindividual variability in the relation between carbohydrates/glycemic load and PPGR characteristics (iAUC120 and BGRise). These data may explain that the small impact of GL in developed models is due to the high interpatient variability of PPGR and confirms the concept that individual responses to GI value determinations might vary dramatically in different patients [30]. Another reason might be a high within-subject variability of PPGR [30].

Experts in GI methodology recognize that “within-individual variation does influence the accuracy and precision of measured GI values, and for this reason, GI methodology has been designed to minimize these effects”; namely, “the denominator in the GI calculation must be the mean of ≥2 tests of the reference food in each subject” [31]. To minimize the intra-individual coefficient of variation (CV), it is recommended by the International Standards Organization that the GI value of the test food be derived from the ratio of the glycemic response it elicits over an average of two, preferably three, glycemic responses to the reference [32]. However, in several studies, intra-individual variability was not reduced with this testing strategy [30,33,34]. Thus, despite using recommended GI methodology, Matthan et al. documented substantial variability in the mean intra-individual (20%) and interindividual (25%) CVs for a single food (white bread) [30].

One of the core limitations of the presented study is the self-report nature of the dietary data. Particularly, GDM patients could omit reporting intake of “forbidden” products (e.g., sweets) and misreport portion sizes. Some of the participants did not reliably record the time of food intake; it could be that some of that misreporting could not be detected with automated algorithms based on thresholds used in the study. This is a typical drawback of any study assessing nutrition in free-living conditions without feeding participants.

Another important reason that may introduce bias into the study data is the fact that GI values of the “same” food as given in the International GI Tables may vary widely for some foods [35]. Consequently, it is impossible to know the exact GI value of the specific food a research subject is actually eating. This makes the use of GI less accurate than it could be for PPGR prediction in free-living conditions. However, the glycemic responses even to specific foods have been shown to have significant intra- and interindividual variability [6,8,30].

Additionally, the GI values of foods in our nutrient databases may be not accurate enough because it was assigned according to published GI data and not directly measured for each food item. Indeed, it was shown that calculated diet GI values may differ substantially depending on who created the GI database, because different people might ascribe different GI values to the same food items [36]. However, it is a well-recognized problem facing all nutritional studies performed in free-living conditions: the challenge of providing reliable GI data for specific foods to consumers and health professionals.

Moreover, GI values assigned to our food database were derived only from studies performed on healthy individuals, while the GI values obtained from diabetes (DM) patients were not included. There is no published database of GI values obtained from pregnant women with GDM. However, this population of women has a kind of intermediate impairment in glucose tolerance, placing them between healthy individuals and “overt” DM patients. Thus, the GI values obtained from healthy volunteers may be not precise enough for women with GDM. In the study by Matthan et al. longer-term glycemic control as reflected by HbA1c values was an important contributor to the variability of GI even in subjects without diabetes [30]. Studies in individuals with normal and impaired glucose tolerance and DM [37,38] led to the conclusion that glycemic status does not significantly affect the mean GI value, even though the variability differs among groups. However, it was recommended that GI values be determined in normoglycemic individuals [21].

In spite of the high inter- and intrapersonal variability of PPGR to the same food, the use of GI data for guiding dietary recommendations has been shown to have significant albeit modest beneficial effects on different health outcomes. Low GI diets have been shown to improve glycemic control [39,40,41], to reduce calculated coronary heart disease (CHD) risk score, to decrease interleukin-6 [42] in people with diabetes, to improve maintenance of weight loss [43], and to considerably reduce diurnal glycemic oscillations in women with risk factors for GDM [44]. Thus, there is good reason to believe that incorporation of GI data into dietary general recommendations will improve a number of health outcomes, but the use of GI/GL data did not considerably increase the accuracy of individual PPGR prediction, which could be used to further improve pregnancy outcomes through personalized nutrition.

The correlation between the predicted and observed values of PPGR obtained in our study (*R* = 0.584 for iAUC120) was modest compared to the value of 0.7 observed for the Israeli population. However, it was close to the correlation obtained in the study in USA: *R* = 0.596 and *R* = 0.618 depending on the number of individuals on which the algorithm was trained [8]. Of note, the degree of reproducibility (best possible predictive performance) in the US population was 0.660 observed using standardized meals [8]. It is also important to mention that those studies implemented a more complicated gradient boosting of regression trees in comparison to generalized linear models utilized in our study, which might have resulted in the overall higher correlation between the predicted and observed values, as those models can describe more complex patterns in data. Evaluation of gradient boosting models for data presented in the study will be held in the following study.

The precision acquired for iAUC120 and BGRise predictive models implies that some other factors could be more important than those examined in the study. Promising ways to increase the accuracy of PPGR prediction models include adding data on physical activity [45], gut microbiome [6,7], and genetics [46].

As there is a non-linear relation between BG levels and meal composition, more complicated models should be examined. The study also shows the limits to which extent linear models could be utilized to predict PPGR. Future research directions include the addition of physical activity and sleep monitoring by means of fitness bracelets. More complicated models, e.g., neural networks and stochastic gradient boosting regression, or ensembles of models will be examined.

## 5. Conclusions

Inclusion of GI into a food database and into PPGR predictive models did not substantially increase the accuracy of individual PPGR prediction. In our study performed in free-living conditions, the amount of carbohydrates was a more important contributor to regression models than GL and GI. The small impact of GI/GL into the individual PPGR may be explained by the substantial variability in individual responses to GI value determinations [30] and intraindividual variability of PPGR to specific foods [6,8].

Furthermore, some criticisms cast doubt upon the usefulness of GI for PPGR prediction, asserting that it is difficult to implement GI and GL in clinical practice when there are different combinations and proportions of food, because both methods are based on the assessment of PPGR to certain kinds of food [47] and the calculated GI of mixed meals does not coincide with their measured GI [48,49].

However, as almost half of the participants had a higher correlation of PPGR with GL than with the amount of carbohydrates consumed, it may explain the effect of low GI diet in the treatment of DM. It makes sense to include GI data in general dietary recommendations for pregnant women, keeping in mind that not all of them may benefit from using GI data. Further research is needed to explore the ability of more complicated models taking into consideration different individual features to increase the accuracy of PPGR prediction for personalized nutrition recommendations.

## Figures and Tables

**Table 1:** Characteristics of participants.

Characteristic	GDM (*N* = 90)	Control (*N* = 34)	*p*-Value (Two-Sided Test)
Age, years	31.8 ± 4.5	30.5 ± 4.4	0.169
Pre-pregnancy BMI, kg/m	25.6 ± 5.9	22.0 ± 3.7	0.002
HbA1C (%)	5.1 ± 0.4	5.7 ± 0.4	<0.001
Gestational age, week	25.8 ± 4.9	27.3 ± 2.9	0.019
BP systolic, mm Hg	121.8 ± 12.2	115.9 ± 15.5	0.129
BP diastolic, mm Hg	76.6 ± 9.1	73.3 ± 11.9	0.102
Arterial hypertension N (%)	9 (10)	1 (3)	0.286
OGTT Fasting PG, mmol/L	5.2 ± 0.5	4.4 ± 0.4	<0.001
OGTT 1-h PG, mmol/L	9.6 ± 1.7	6.6 ± 1.4	<0.001
OGTT 2-h PG, mmol/L	8.5 ± 2.0	6.0 ± 1.1	<0.001
Fasting serum insulin, pmol/L	92.5 ± 42.4	78.5 ± 54.4	0.132
Fasting leptin, ng/mL	36.7 ± 31.4	33.0 ± 27.6	0.549
Total cholesterol (mmol/L)	6.3 ± 1.2	6.1 ± 1.1	0.306
Triglycerides (mmol/L)	2.1 ± 0.8	1.7 ± 0.7	0.007
HDL-C (mmol/L)	2.0 ± 0.4	2.1 ± 0.4	0.236
LDL-C (mmol/L)	3.4 ± 0.9	3.3 ± 1.0	0.887

BMI—body mass index; HbA1c—hemoglobin A1c; PG—plasma glucose; OGTT—oral glucose tolerance test; BP—blood pressure; GDM—gestational diabetes mellitus, HDL-C—high-density lipoprotein-cholesterol; LDL-C—low-density lipoprotein-cholesterol.

**Table 2:** Correlation between meal characteristics and PPGR.

	Gi	Gl	Carbo	Prot	Fat	Kcal	Water	Starch	Fiber	iAUC120	BG Rise
GI	1	0.406	0.318	−0.141	−0.028 ^b^	0.081	−0.033 ^b^	0.382	0.084	0.199	0.212
GL	0.406	1	0.952	0.112	0.262	0.611	0.285	0.819	0.319	0.423	0.424
carbo	0.318	0.952	1	0.172	0.298	0.670	0.340	0.795	0.400	0.434	0.425
prot	−0.143	0.112	0.172	1	0.527	0.634	0.272	0.129	0.153	0.023 ^b^	0.001 ^b^
fat	−0.028 ^b^	0.262	0.298	0.527	1	0.863	0.232	0.257	0.130	0.078	0.058 ^a^
kcal	0.081	0.611	0.670	0.634	0.863	1	0.378	0.541	0.298	0.248	0.225
water	−0.033 ^b^	0.285	0.340	0.272	0.232	0.378	1	0.235	0.189	0.178	0.188
starch	0.382	0.819	0.795	0.129	0.257	0.541	0.235	1	0.258	0.365	0.366
fiber	0.084	0.319	0.400	0.153	0.130	0.298	0.189	0.258	1	0.135	0.131
iAUC120	0.199	0.423	0.434	0.023 ^b^	0.078	0.248	0.178	0.365	0.135	1	0.945
BGRise	0.212	0.423	0.425	0.001 ^b^	0.058 ^a^	0.225	0.188	0.366	0.131	0.945	1

carbo—carbohydrates, prot—proteins. All correlations except where highlighted are significant on the 0.01 level (two-sided); ^a^—correlation is significant on the level 0.05; ^b^—correlation is not significant.

**Table 3:** Stepwise-regression for predicting glycemic response (iAUC120) for models constructed with available GL and GI features.

Model	*R*	*R* Squared	Adj. *R* Squared	Standard Error
1	0.434 ^a^	0.188	0.188	0.589
2	0.507 ^b^	0.257	0.256	0.564
3	0.531 ^c^	0.282	0.280	0.555
4	0.550 ^d^	0.303	0.301	0.547
5	0.563 ^e^	0.317	0.315	0.541
6	0.573 ^f^	0.329	0.326	0.537
7	0.581 ^g^	0.337	0.334	0.534

^a^ Predictors: (constant) and carbo; ^b^ Predictors: (constant), carbo, and BG0; ^c^ Predictors: (constant), carbo, BG0, and after_1 h_test; ^d^ Predictors: (constant), carbo, BG0, after_1 h_test, and types_food_1; ^e^ Predictors: (constant), carbo, BG0, after_1 h_test, types_food_1, and meat1_2; ^f^ Predictors: (constant), carbo, BG0, after_1 h_test, types_food_1, meat1_2, and sousages1_2; ^g^ Predictors: (constant), carbo, BG0, after_1 h_test, types_food_1, meat1_2, sousages1_2, and N_abortions.

**Table 4:** Coefficients of the linear model on every step of stepwise regression algorithm.

Model		Non-Stand. Coefficients		Stand. Coef.	*t*	Significance
id	variables	B	St. Error	Betta		
a	(constant)	0.415	0.025		16.668	<0.001
	carbo	0.010	0.001	0.434	18.579	<0.001
b	(constant)	10.838	0.123		14.883	<0.001
	carbo	0.010	0.001	0.410	18.238	<0.001
	BG0	−0.277	0.024	−0.264	−11.741	<0.001
c	(constant)	10.352	0.139		9.697	<0.001
	carbo	0.011	0.001	0.452	19.743	<0.001
	BG0	−0.280	0.023	−0.266	−12.057	<0.001
	after_1 h_test	0.054	0.008	0.162	7.091	<0.001

carbo—сarbohydrates; BG0—blood glucose level before food intake; after_1 h_test—plasma glucose level 1 hour after oral glucose tolerance test. ^a^ Predictors: (constant) and carbo; ^b^ Predictors: (constant), carbo, and BG0; ^c^ Predictors: (constant), carbo, BG0, and after_1 h_test.

**Table 5:** Final models predicting different PPGR characteristics selected with stepwise regression.

Model	With GI/GL		Without GI/GL	
	N Coefficients	*R*	N Coefficients	*R*
iAUC120	53	0.705	44	0.700
BGRise	57	0.705	59	0.696
BG60	40	0.700	42	0.698
BGMax	59	0.745	59	0.738
AUC120	53	0.789	44	0.785
iAUC60	50	0.836	50	0.833
AUC60	50	0.658	50	0.651

**Table 6:** Results of prediction on the test set.

Model	N Coefficients	*R* Test	MAE Test	Inclusion of GI/GL *
iAUC120	2	0.564	0.455	no
BGRise	4	0.524	0.700	no
BG60	4	0.517	0.673	no
BGMax, with GI/GL	4	0.519	0.695	yes
BGMax, without GI/GL	3	0.520	0.700	
AUC120, with GI/GL	11	0.653	0.453	yes
AUC120, without GI/GL	4	0.643	0.448	
iAUC60, with GI/GL	5	0.462	0.385	yes
iAUC60, without GI/GL	4	0.481	0.383	
AUC60, with GI/GL	2	0.734	0.385	no

* Inclusion of GI/GL by regularized regression algorithm.

**Table 7:** Results of prediction on the test set with added polynomic features.

Model	N Coefficients	*R* Test	MAE Test
iAUC120, with GI/GL	7	0.584	0.447
iAUC120, without GI/GL	7	0.584	0.446
BGRise, with GI/GL	19	0.554	0.680
BGRise, without GI/GL	13	0.551	0.680
BG60, with GI/GL	6	0.535	0.665
BG60, without GI/GL	5	0.533	0.665
BGMax, with GI/GL	10	0.549	0.681
BGMax, without GI/GL	9	0.548	0.689
AUC120, with GI/GL	9	0.673	0.446
AUC120, without GI/GL	6	0.675	0.442
iAUC60, with GI/GL	6	0.464	0.383
iAUC60, without GI/GL	7	0.495	0.475
AUC60, with GI/GL	7	0.750	0.374
AUC60, without GI/GL	5	0.750	0.375

## Supplementary Materials

The following are available online at https://www.mdpi.com/2072-6643/12/2/302/s1. Figure S1: Examples of CGM and meal data from patients with different quality of meal diaries. List S2: The complete list of features added as inputs for the blood glucose models. Figure S3: Individual correlation between meal data and PPGR for patients with different correlation. Table S4: The complete list of coefficients for stepwise regression model built with least-square algorithm on a full dataset. List S5: Resulting formulas for PPGR predictive models.
