# Supplementary material for: The Role of Glycemic Index and Glycemic Load in the Development of Real-Time Postprandial Glycemic Response Prediction Models for Patients with Gestational Diabetes
Source: Nutrients. 2020 Jan 23;12(2):302. doi: 10.3390/nu12020302 (PMC7071209; doi:10.3390/nu12020302)
Supplement: Supplementary file 1 [file nutrients-12-00302-s001.pdf]

Supplementary materials

S1. Examples of CGM and meal data from patients with different quality of meal diaries.

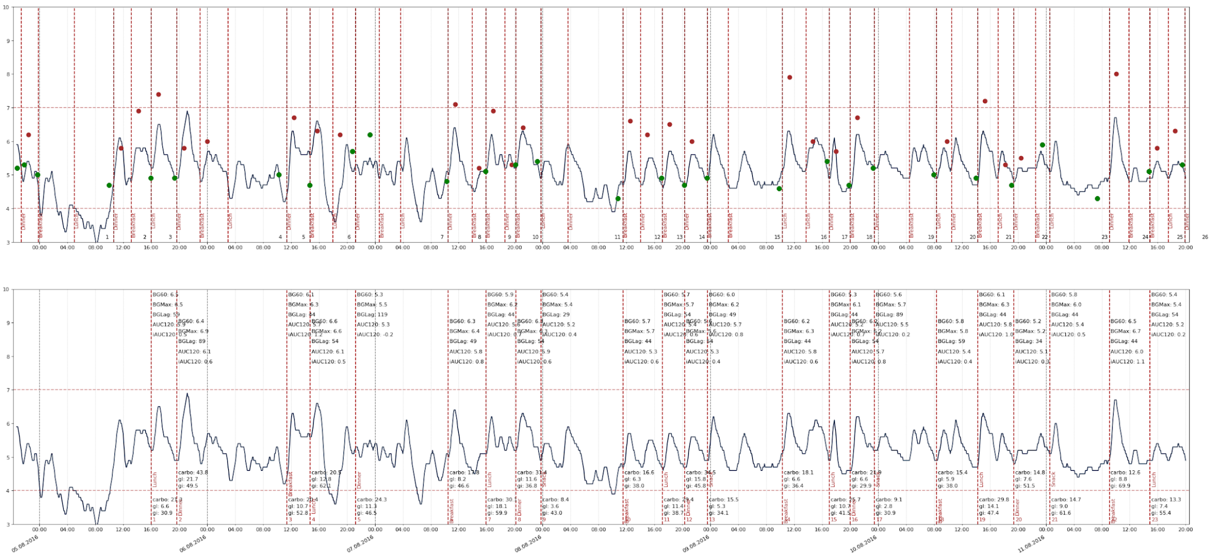

(**a**)

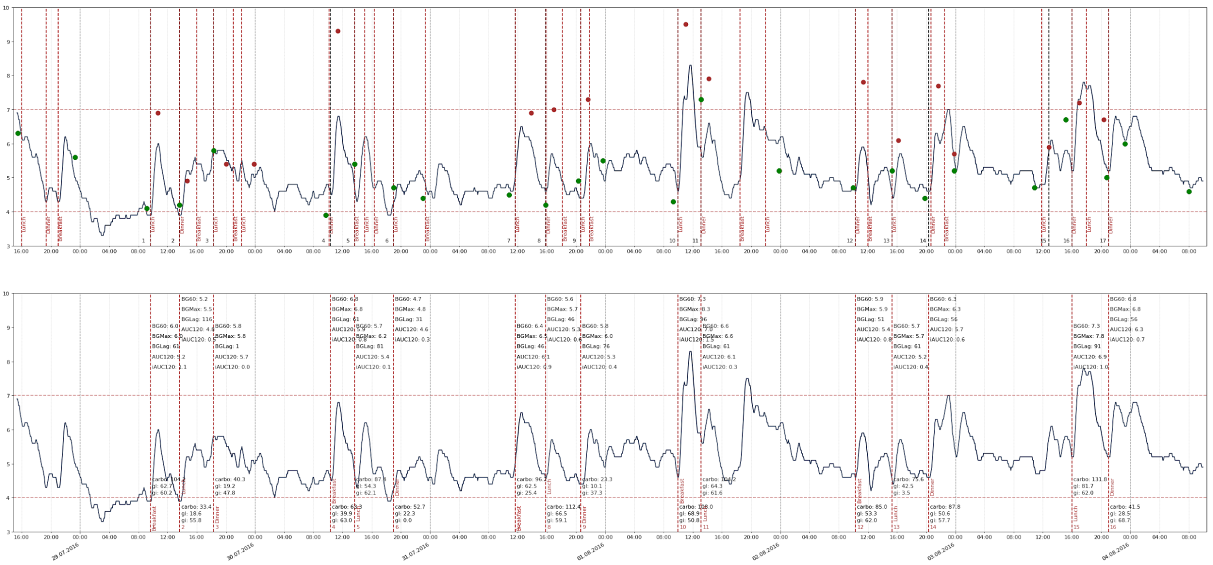

(**b**)

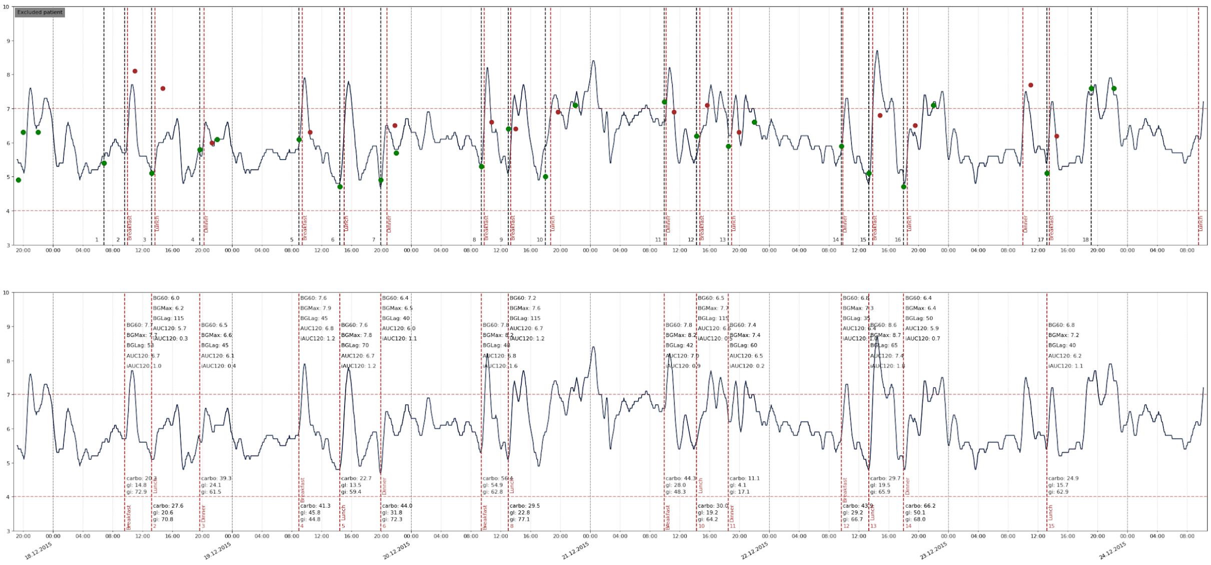

(c)

Figure S1. CGM and food diary data from a patient with good (**a**), mediocre (**b**) and bad (**c**) quality of meal diary. On the top: black vertical lines - meal starts written in a paper protocol, green vertical lines - meal ends written in the paper protocol, red line - meal starts as written in the electronic diary; on the bottom: red lines - meal starts chosen for the final sets, upper marks - PPGR features, lower marks - meal features. Green dots represent point estimations of BG levels made with glucometer used for sensor calibration and red points correspond to point estimations 1 hour after the meal.

S2. The complete list of features added as inputs for the blood glucose models.

group – group number (1 – GDM, 2 – healthy)

n_cgm – the order of CGMS installation (1 - first, 2 - second)

preg_week – gestational age at the day of the meal

types_food – meal type (1 - breakfast, 2 - lunch, 3 - dinner, 4 - snack)

gi – glycemic index of the meal

gl – glycemic load of the meal

carbo – the amount of carbohydrates in the meal, in g

prot – the amount of proteins in the meal, in g

fat – the amount of fats in the meal, in g

kkal – the energy value of the meal

water – the amount of water in the meal, in g

mds – the amount of monosaccharides and disaccharide in the meal, in g

kr – the amount of starch in the meal, in g

pv – the amount of alimentary fiber in the meal, in g

ok – the amount of organic acids in the meal, in g

zola – the amount of ash in the meal, in g

na – the amount of Na in the meal, in mg

k – the amount of K in the meal, in mg

ca – the amount of Ca in the meal, in mg

mg – the amount of Mg in the meal, in mg

p – the amount of P in the meal, in mg

fe – the amount of iron in the meal, in mg

a – the amount of retinol in the meal, in mcg

kar – the amount of beta-carotene in the meal, in mcg

re – the amount of retinol equivalent in the meal, in mcg

b1 – the amount of thiamine in the meal, in mg

b2 – the amount of riboflavin in the meal, in mg

ne – the amount of niacin equivalent in the meal, in mg

c – the amount of ascorbic acid, in mg

BG0 – blood glucose level at the beginning of the meal on CGM signal

HbA1C_V1 – glycosylated hemoglobin at the time of inclusion into the study

N_abortions – the number of abortions in history

N_pregnancies – the number of pregnancies in history

N_pregnancy_loss – the number of pregnancy loss episodes in history

N_deliveries – the number of deliveries in history

AH – arterial hypertension

BP_dyast1 – diastolic blood pressure at the time of inclusion into the study, mm Hg

BP_syst1 – systolic blood pressure at the time of inclusion into the study, mm Hg

beta_OHB_V1 – beta-hydroxybutyrate at the time of inclusion into the study

Fasting_PG – fasting plasma glucose at the time of inclusion into the study

BMI – prepregnancy body mass index, kg/m2

AI_V1 – atherogenic index at the time of inclusion into the study (V1)

COC – combined oral contraceptives use (1 – yes, 0 – no)

HDLC_V1 – high density lipoprotein cholesterol at the time of inclusion into the study

LDLC_V1 – low density lipoprotein cholesterol at the time of inclusion into the study

VLDLC_V1 – very low density lipoprotein cholesterol at the time of inclusion into the study

IGT – impaired glucose tolerance before pregnancy

TG_V1 – serum triglyceride level (mmol/L) at the time of inclusion into the study

FR_V1 – serum fructosamine level (mcmol/l) at the time of inclusion into the study

Chol_V1 – cholesterol level (mmol/L) at the time of inclusion into the study

Weight – prepregnancy weight, kg

Age – age, years

Diet_start – gestational age at the time of starting of dieting

insulin_V1 – plasma insulin level at the time of inclusion into the study

ketones_V1 – urinary ketones level at the time of inclusion into the study

leptin_V1 – serum leptin (ng/ml) level at the time of inclusion into the study

FPG_OGTT – fasting plasma glucose in OGTT

height – height, cm

gest_age_V1 – gestational age at the time of testing V1 (at the time of inclusion into the study)

GA_sm_stopped – gestational age when smoking was stopped

smoking_duration – smoking duration, years (before pregnancy)

PG_1h – 1-hour plasma glucose level in OGTT

PG_2h – 2-hours plasma glucose level in OGTT

menses – regularity of menstrual cycle (1 – yes, 0 – no)

CI – cervical insufficiency (1 – yes, 2 – no)

DM_hystory – diabetes mellitus in family history (1 – yes, 0 - no)

PCOS – polycystic ovary syndrome (0 - no, 1 – yes)

alcohol1 – alcohol consumption before pregnancy (1- no alcohol consumption before pregnancy; 2- alcohol consumption before pregnancy 0.5 - 2 times/week; 3-alcohol consumption before pregnancy more than 2 times/week)

alcohol2 – alcohol consumption during pregnancy (1- no alcohol consumption before pregnancy; 2- alcohol consumption before pregnancy 0.5 - 2 times/week; 3-alcohol consumption before pregnancy more than 2 times/week)

legumes1 – legumes consumption before pregnancy (1- less than 1 time per week; 2- 1-3 times a week; 3- more than 3 times a week)

legumes2 – legumes consumption during pregnancy (1- less than 1 time per week; 2- 1-3 times a week; 3- more than 3 times a week)

pastries1 – eating pastries before pregnancy (1- less than 2 per week; 2- 2-4 times a week; 3- more than 4 times a week)

pastries2 – eating pastries during pregnancy (1- less than 2 times per week; 2- 2-4 times a week; 3- more than 4 times a week)

GDM_history – GDM in history (1 – yes, 2 – no)

coffee1 – drinking coffee before pregnancy (1- 0-1 per day; 2- 2-3 per day; 3 -more than 3 times per day)

coffee2 – drinking coffee during pregnancy (1- 0-1 cup per day; 2- 2-3 per day; 3 -more than 3 times per day)

smoking_2 – smoking during pregnancy (0- no, 1 – yes)

smoking_before – smoking before pregnancy (0- no, 1 – yes)

dairy_products1 – dairy products before pregnancy (1- less than 3 times per week; 2- 3-6 times a week; 3 - more than 6 times a week)

dairy_products2 – dairy products during pregnancy (1- less than 3 per week; 2- 3-6 times a week; 3 -more than 6 times a week)

skimmed_dairy_products1 – skimmed dairy foods before pregnancy (1- less than 3 times per week; 2- 3-6 times a week; 3 -more than 6 times a week)

skimmed_dairy_products2 – skimmed dairy foods during pregnancy (1- less than 3 times per week; 2- 3-6 times a week; 3 -more than 6 times a week)

meat1 – eating meat before pregnancy (1- less than 3 times a week; 2 -3-6 times a week; 3 - more than 6 times a week)

meat2 – eating meat during pregnancy (1- less than 3 times a week; 2 -3-6 times a week; 3 - more than 6 times a week)

education – level of education: 1- secondary, 2 - higher

vegetables1 – eating vegetables before pregnancy (1- less than 6 times per week; 2- 6-12 times a week; 3 - more than 12 times a week)

vegetables1_raw – eating raw vegetables before pregnancy (1- less than 6 per week; 2- 6-12 times a week; 3 -more than 12 times a week)

vegetables2 – eating vegetables during pregnancy (1- less than 6 per week; 2- 6-12 times a week; 3 -more than 12 times a week)

vegetables2_raw – eating raw vegetables during pregnancy (1- less than 6 per week; 2- 6-12 times a week; 3 -more than 12 times a week)

edema1 – edema during pregnancy (0 – no, 1 - yes)

cakes1 – eating cakes before pregnancy (1- less than 2 time per week; 2- 2-4 times a week; 3- more than 4 times a week)

cakes2 – eating cakes during pregnancy (1- less than 2 time per week; 2- 2-4 times a week; 3- more than 4 times a week)

climbing_the_stairs1 – the amount of walking up the stairs before pregnancy (1- less than 4 flights per day; 2 -4-16 flights of stairs per day; 3 -more than 16 flights of stairs per day)

climbing_the_stairs2 – the amount of walking up the stairs during pregnancy (1- less than 4 flights per day; 2 -4-16 flights of stairs per day; 3 -more than 16 flights of stairs per day)

placenta_previa – placenta previa during pregnancy (1 – yes, 0 – no)

prolactin – history of hyperprolactinemia (1 – yes, 0 – no)

fish1 – frequency of fish consumption before pregnancy (1- less than 3 times per week; 2- 3-6 times a week; 3 -more than 6 times a week)

fish2 – frequency of fish consumption during pregnancy (1- less than 3 times per week; 2- 3-6 times a week; 3 -more than 6 times a week)

sweet_drinks1 – drinking sweet drinks before pregnancy (1- less than 2 times per week; 2 -2-4 times a week; 3 - more than 4 times a week)

sweet_drinks2 – drinking sweet drinks during pregnancy (1- less than 2 times per week; 2 -2-4 times a week; 3 - more than 4 times a week)

sausages1 – frequency of consumption of sausage products before pregnancy (1 - less than 1 time a week; 2 - 1-3 times a week; 3 -more than 3 times a week)

sausages2 – frequency of sausage products consumption during pregnancy (1 - less than 1 time per week; 2 - 1-3 times a week; 3 -more than 3 times a week)

sauces1 – use of sauces before pregnancy (1- less than 2 times per week; 2 - 2-4 times a week; 3- more than 4 times a week)

sauces2 – use of sauces during pregnancy (1- less than 2 times per week; 2 - 2-4 times a week; 3- more than 4 times a week)

performing_sports1 – performing sports before pregnancy (1- less than 2 times a week; 2- 2-3 times a week; 3 - more than 3 times a week)

performing_sports2 – performing sports during pregnancy (1- less than 2 times a week; 2- 2-3 times a week; 3 - more than 3 times a week)

dried_fruits_1 – dried fruit before pregnancy (1-0; 2- 1-3 times a week; 3 - more than 3 times a week)

dried_fruits_2 – dried fruit during pregnancy (1-0; 2- 1-3 times a week; 3 - more than 3 times a week)

threatened_miscarriage – threatened miscarriage at any time of pregnancy (1 – yes, 0 – no)

fruits1 – eating fruits before pregnancy (1- less than 6 per week; 2 - 6-12 per week; 3 - more than 12 per week)

fruits2 – eating fruits during pregnancy (1- less than 6 per week; 2 - 6-12 per week; 3 - more than 12 per week)

bread_any1 – eating bread (any) before pregnancy (1- less than 6 times per week; 2- 6-12 times a week; 3 - more than 12 times per week)

bread_any2 – eating bread (any) during pregnancy (1- less than 6 times per week; 2- eating 6-12 times a week; 3 - more than 12 times per week)

bread_whole_grain_bread1 – eating whole grain bread before pregnancy (1- less than 1 time per week; 2- less than 1-3 times a week; 3 -more than 3 times a week)

bread_whole_grain_bread2 – eating whole grain bread during pregnancy (1- less than 1 time per week; 2- less than 1-3 times a week; 3 -more than 3 times a week)

walking1 – walking before pregnancy (1- less than 30 minutes a day; 2- 30-60 minutes a day; 3 -more than 60 minutes a day)

walking2 – walking during pregnancy (1- less than 30 minutes a day; 2- 30-60 minutes a day; 3 -more than 60 minutes a day)

chocolate1 – eating chocolate before pregnancy (1- less than 2 times a week; 2- 2-4 times a week; 3- more than 4 times a week)

chocolate2 – eating chocolate during pregnancy (1- less than 2 times a week; 2- 2-4 times a week; 3- more than 4 times a week)

AUC120 – area under the glycaemic curve 2 hours after the start of the meal

AUC60 – area under the glycaemic curve 1 hour after the start of the meal

BG60 – BG 60 minutes after the start of food intake

BGMax – peak blood glucose level during 3 hours after the meal

BGRise – the rise of blood glucose level from the beginning of the meal to the peak value

iAUC120 – incremental area under the glycaemic curve 2 hours after food intake

iAUC60 – incremental area under the glycaemic curve 1 hour after food intake

S3. Individual correlation between meal data and PPGR for patients with different correlation

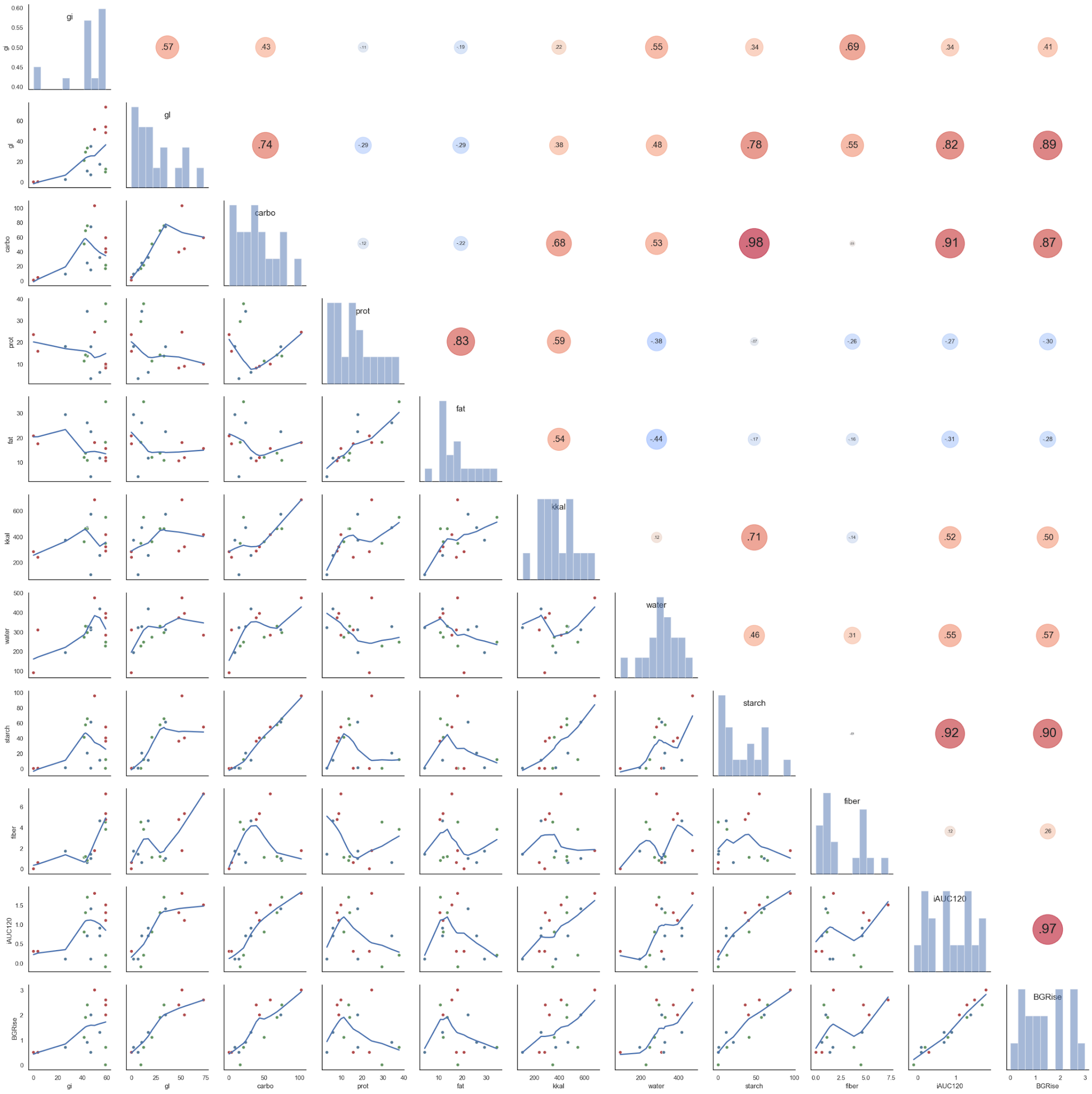

(**a**)

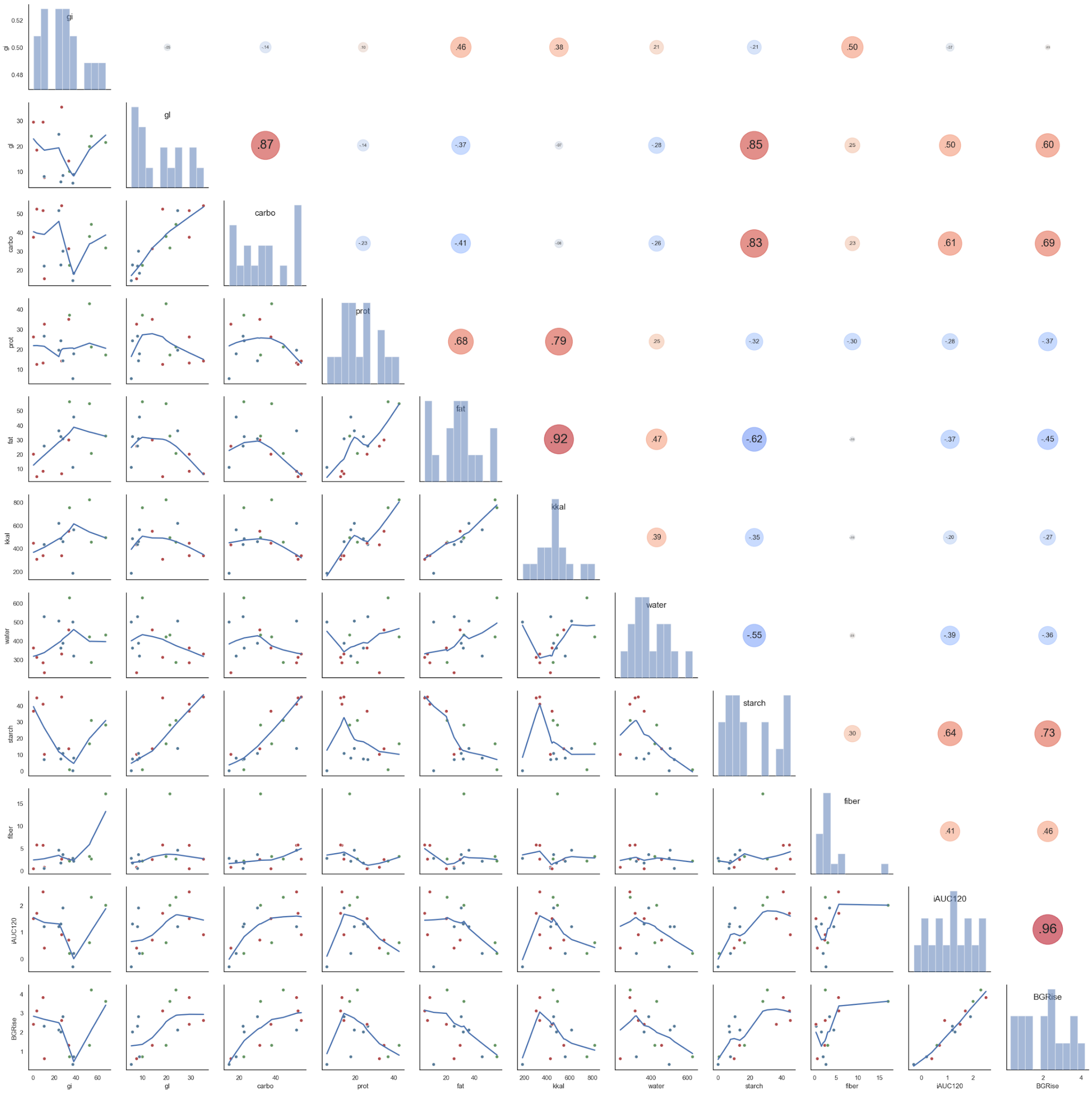

gi
gl
carbo
prot
fat
kkal
water
starch
fiber
iAUC120
BGRise
.87
.85
.83
.92
.96
.73
.69
.79
.68
-.62
.64
.61
.60
-.55
.50
.50
.47
.46
.46
.41
-.41

(b)

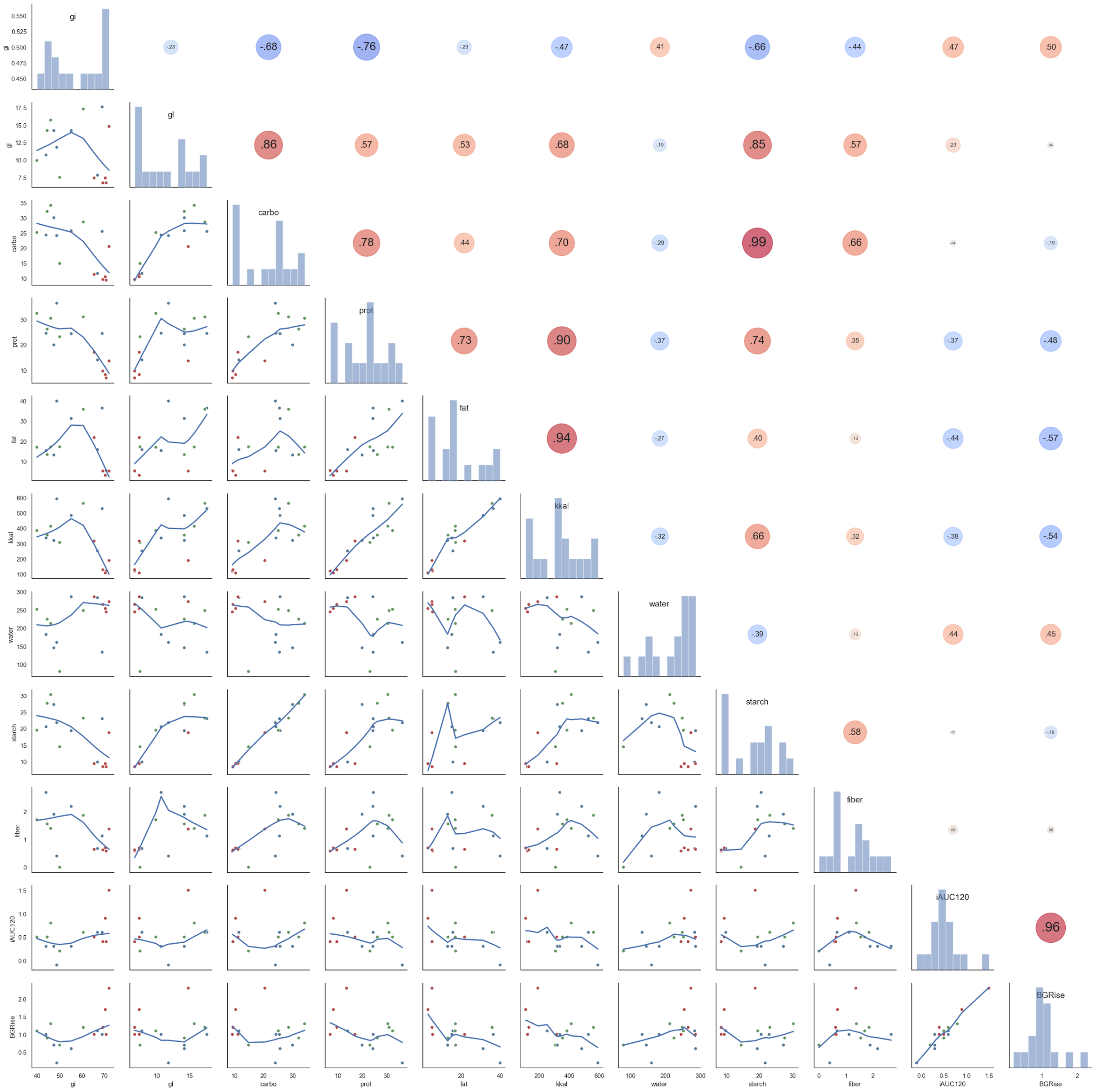

(c)

Figure S3. The relation between key characteristics, that were chosen for data analysis for a single patient with high (a), mediocre (b) and low (c) correlation between glycemic load and PPGR. Upper-ring corner: coefficients of correlation, diagonal: histograms for each value, lower-left corner: scatter plots and regression curve; colors depict meal type: breakfast - red, lunch - blue, dinner - green, snack - purple.

S4. The complete list of coefficients for linear models built via stepwise regression with least-square algorithm on a full dataset.

Model	R	R-squared	Adjusted R-κsquared	Standard error of estimation
1	.434[a]	.189	.188	.5897
2	.507[b]	.257	.256	.5644
3	.529[c]	.280	.279	.5558
4	.549[d]	.302	.300	.5476
5	.564[e]	.318	.316	.5414
6	.574[f]	.330	.327	.5368
7	.586[g]	.344	.341	.5314
8	.593[h]	.352	.349	.5281
9	.600[i]	.360	.357	.5249
10	.607[j]	.368	.364	.5219
11	.615[k]	.378	.374	.5179
12	.622[l]	.387	.382	.5146
13	.629[m]	.396	.391	.5107
14	.636[n]	.405	.399	.5073
15	.641[o]	.410	.404	.5050
16	.645[p]	.416	.410	.5026
17	.650[q]	.423	.416	.5001
18	.655[r]	.429	.422	.4977
19	.658[s]	.433	.426	.4959
20	.661[t]	.437	.430	.4942
21	.664[u]	.441	.433	.4928
22	.667[v]	.445	.437	.4910
23	.670[w]	.449	.440	.4896
24	.672[x]	.452	.443	.4883
25	.674[y]	.455	.446	.4872
26	.677[z]	.458	.449	.4859
27	.679[aa]	.460	.451	.4851
28	.680[ab]	.463	.453	.4841
29	.682[ac]	.465	.455	.4833

30	.684[ad]	.467	.456	.4825
31	.685[ae]	.470	.458	.4816
32	.685[af]	.469	.458	.4816
33	.687[ag]	.472	.461	.4804
34	.689[ah]	.474	.463	.4796
35	.690[ai]	.476	.465	.4789
36	.692[aj]	.479	.466	.4780
37	.694[ak]	.481	.469	.4770
38	.696[al]	.484	.471	.4759
39	.697[am]	.486	.473	.4751
40	.700[an]	.489	.476	.4737
41	.699[ao]	.489	.476	.4739
42	.701[ap]	.491	.478	.4730
43	.702[aq]	.493	.479	.4722
44	.704[ar]	.495	.481	.4714
45	.703[as]	.494	.481	.4716
46	.705[at]	.497	.483	.4705
47	.707[au]	.500	.486	.4693
48	.708[av]	.501	.487	.4687
49	.709[aw]	.503	.488	.4682
50	.710[ax]	.505	.490	.4674

a. Predictors: (intercept), carbo

b. Predictors: (intercept), carbo, BG0

c. Predictors: (intercept), carbo, BG0, PG_1h

d. Predictors: (intercept), carbo, BG0, PG_1h, types_food_n_1

e. Predictors: (intercept), carbo, BG0, PG_1h, types_food_n_1, HDLC_V1

f. Predictors: (intercept), carbo, BG0, PG_1h, types_food_n_1, HDLC_V1, climbing_the_stairs1_3

g. Predictors: (intercept), carbo, BG0, PG_1h, types_food_n_1, HDLC_V1, climbing_the_stairs1_3, dairy_products2_3

h. Predictors: (intercept), carbo, BG0, PG_1h, types_food_n_1, HDLC_V1, climbing_the_stairs1_3, dairy_products2_3, chocolate1_2

i. Predictors: (intercept), carbo, BG0, PG_1h, types_food_n_1, HDLC_V1, climbing_the_stairs1_3, dairy_products2_3, chocolate1_2, whole_grain_bread_1_1

j. Predictors: (intercept), carbo, BG0, PG_1h, types_food_n_1, HDLC_V1, climbing_the_stairs1_3, dairy_products2_3, chocolate1_2, whole_grain_bread_1_1, alcohol2_1

k. Predictors: (intercept), carbo, BG0, PG_1h, types_food_n_1, HDLC_V1, climbing_the_stairs1_3, dairy_products2_3, chocolate1_2, whole_grain_bread_1_1, alcohol2_1, coffee2_1

l. Predictors: (intercept), carbo, BG0, PG_1h, types_food_n_1, HDLC_V1, climbing_the_stairs1_3, dairy_products2_3, chocolate1_2, whole_grain_bread_1_1, alcohol2_1, coffee2_1, DM_hystory_0

m. Predictors: (intercept), carbo, BG0, PG_1h, types_food_n_1, HDLC_V1, climbing_the_stairs1_3, dairy_products2_3, chocolate1_2, whole_grain_bread_1_1, alcohol2_1, coffee2_1, DM_hystory_0, placenta_previa_0

n. Predictors: (intercept), carbo, BG0, PG_1h, types_food_n_1, HDLC_V1, climbing_the_stairs1_3, dairy_products2_3, chocolate1_2, whole_grain_bread_1_1, alcohol2_1, coffee2_1, DM_hystory_0, placenta_previa_0, gi

o. Predictors: (intercept), carbo, BG0, PG_1h, types_food_n_1, HDLC_V1, climbing_the_stairs1_3, dairy_products2_3, chocolate1_2, whole_grain_bread_1_1, alcohol2_1, coffee2_1, DM_hystory_0, placenta_previa_0, gi, Chol_V1

p. Predictors: (intercept), carbo, BG0, PG_1h, types_food_n_1, HDLC_V1, climbing_the_stairs1_3, dairy_products2_3, chocolate1_2, whole_grain_bread_1_1, alcohol2_1, coffee2_1, DM_hystory_0, placenta_previa_0, gi, Chol_V1, vegetables1_2

q. Predictors: (intercept), carbo, BG0, PG_1h, types_food_n_1, HDLC_V1, climbing_the_stairs1_3, dairy_products2_3, chocolate1_2, whole_grain_bread_1_1, alcohol2_1, coffee2_1, DM_hystory_0, placenta_previa_0, gi, Chol_V1, vegetables1_2, cakes2_1

r. Predictors: (intercept), carbo, BG0, PG_1h, types_food_n_1, HDLC_V1, climbing_the_stairs1_3, dairy_products2_3, chocolate1_2, whole_grain_bread_1_1, alcohol2_1, coffee2_1, DM_hystory_0, placenta_previa_0, gi, Chol_V1, vegetables1_2, cakes2_1, group_1

s. Predictors: (intercept), carbo, BG0, PG_1h, types_food_n_1, HDLC_V1, climbing_the_stairs1_3, dairy_products2_3, chocolate1_2, whole_grain_bread_1_1, alcohol2_1, coffee2_1, DM_hystory_0, placenta_previa_0, gi, Chol_V1, vegetables1_2, cakes2_1, group_1, meat2_3

t. Predictors: (intercept), carbo, BG0, PG_1h, types_food_n_1, HDLC_V1, climbing_the_stairs1_3, dairy_products2_3, chocolate1_2, whole_grain_bread_1_1, alcohol2_1, coffee2_1, DM_hystory_0, placenta_previa_0, gi, Chol_V1, vegetables1_2, cakes2_1, group_1, meat2_3, water

u. Predictors: (intercept), carbo, BG0, PG_1h, types_food_n_1, HDLC_V1, climbing_the_stairs1_3, dairy_products2_3, chocolate1_2, whole_grain_bread_1_1, alcohol2_1, coffee2_1, DM_hystory_0, placenta_previa_0, gi, Chol_V1, vegetables1_2, cakes2_1, group_1, meat2_3, water, coffee1_1

v. Predictors: (intercept), carbo, BG0, PG_1h, types_food_n_1, HDLC_V1, climbing_the_stairs1_3, dairy_products2_3, chocolate1_2, whole_grain_bread_1_1, alcohol2_1, coffee2_1, DM_hystory_0, placenta_previa_0, gi, Chol_V1, vegetables1_2, cakes2_1, group_1, meat2_3, water, coffee1_1, smok_before_0

w. Predictors: (intercept), carbo, BG0, PG_1h, types_food_n_1, HDLC_V1, climbing_the_stairs1_3, dairy_products2_3, chocolate1_2, whole_grain_bread_1_1, alcohol2_1, coffee2_1, DM_hystory_0, placenta_previa_0, gi, Chol_V1, vegetables1_2, cakes2_1, group_1, meat2_3, water, coffee1_1, smok_before_0, edema1_0

x. Predictors: (intercept), carbo, BG0, PG_1h, types_food_n_1, HDLC_V1, climbing_the_stairs1_3, dairy_products2_3, chocolate1_2, whole_grain_bread_1_1, alcohol2_1, coffee2_1, DM_hystory_0, placenta_previa_0, gi, Chol_V1, vegetables1_2, cakes2_1, group_1, meat2_3, water, coffee1_1, smok_before_0, edema1_0, dried_fruits_1_1

y. Predictors: (intercept), carbo, BG0, PG_1h, types_food_n_1, HDLC_V1, climbing_the_stairs1_3, dairy_products2_3, chocolate1_2, whole_grain_bread_1_1, alcohol2_1, coffee2_1, DM_hystory_0, placenta_previa_0, gi, Chol_V1, vegetables1_2, cakes2_1, group_1, meat2_3, water, coffee1_1, smok_before_0, edema1_0, dried_fruits_1_1, beta_OHB_V1

z. Predictors: (intercept), carbo, BG0, PG_1h, types_food_n_1, HDLC_V1, climbing_the_stairs1_3, dairy_products2_3, chocolate1_2, whole_grain_bread_1_1, alcohol2_1, coffee2_1, DM_hystory_0, placenta_previa_0, gi, Chol_V1, vegetables1_2, cakes2_1, group_1, meat2_3, water, coffee1_1, smok_before_0, edema1_0, dried_fruits_1_1, beta_OHB_V1, bread_any1_2

aa. Predictors: (intercept), carbo, BG0, PG_1h, types_food_n_1, HDLC_V1, climbing_the_stairs1_3, dairy_products2_3, chocolate1_2, whole_grain_bread_1_1, alcohol2_1, coffee2_1, DM_hystory_0, placenta_previa_0, gi, Chol_V1, vegetables1_2, cakes2_1, group_1, meat2_3, water, coffee1_1, smok_before_0, edema1_0, dried_fruits_1_1, beta_OHB_V1, bread_any1_2, pastries1_1

ab. Predictors: (intercept), carbo, BG0, PG_1h, types_food_n_1, HDLC_V1, climbing_the_stairs1_3, dairy_products2_3, chocolate1_2, whole_grain_bread_1_1, alcohol2_1, coffee2_1, DM_hystory_0, placenta_previa_0, gi, Chol_V1, vegetables1_2, cakes2_1, group_1, meat2_3, water, coffee1_1, smok_before_0, edema1_0, dried_fruits_1_1, beta_OHB_V1, bread_any1_2, pastries1_1, sweet_drinks1_1

ac. Predictors: (intercept), carbo, BG0, PG_1h, types_food_n_1, HDLC_V1, climbing_the_stairs1_3, dairy_products2_3, chocolate1_2, whole_grain_bread_1_1, alcohol2_1, coffee2_1, DM_hystory_0, placenta_previa_0, gi, Chol_V1, vegetables1_2, cakes2_1, group_1, meat2_3, water, coffee1_1, smok_before_0, edema1_0, dried_fruits_1_1, beta_OHB_V1, bread_any1_2, pastries1_1, sweet_drinks1_1, preg_week

ad. Predictors: (intercept), carbo, BG0, PG_1h, types_food_n_1, HDLC_V1, climbing_the_stairs1_3, dairy_products2_3, chocolate1_2, whole_grain_bread_1_1, alcohol2_1, coffee2_1, DM_hystory_0, placenta_previa_0, gi, Chol_V1, vegetables1_2, cakes2_1, group_1, meat2_3, water, coffee1_1, smok_before_0, edema1_0, dried_fruits_1_1, beta_OHB_V1, bread_any1_2, pastries1_1, sweet_drinks1_1, preg_week, fish1_3

ae. Predictors: (intercept), carbo, BG0, PG_1h, types_food_n_1, HDLC_V1, climbing_the_stairs1_3, dairy_products2_3, chocolate1_2, whole_grain_bread_1_1, alcohol2_1, coffee2_1, DM_hystory_0, placenta_previa_0, gi, Chol_V1, vegetables1_2, cakes2_1, group_1, meat2_3, water, coffee1_1, smok_before_0, edema1_0, dried_fruits_1_1, beta_OHB_V1, bread_any1_2, pastries1_1, sweet_drinks1_1, preg_week, fish1_3, AI_V1

af. Predictors: (intercept), carbo, BG0, PG_1h, types_food_n_1, HDLC_V1, climbing_the_stairs1_3, dairy_products2_3, chocolate1_2, whole_grain_bread_1_1, alcohol2_1, coffee2_1, DM_hystory_0, placenta_previa_0, gi, vegetables1_2, cakes2_1, group_1, meat2_3, water, coffee1_1, smok_before_0, edema1_0, dried_fruits_1_1, beta_OHB_V1, bread_any1_2, pastries1_1, sweet_drinks1_1, preg_week, fish1_3, AI_V1

ag. Predictors: (intercept), carbo, BG0, PG_1h, types_food_n_1, HDLC_V1, climbing_the_stairs1_3, dairy_products2_3, chocolate1_2, whole_grain_bread_1_1, alcohol2_1, coffee2_1, DM_hystory_0, placenta_previa_0, gi, vegetables1_2, cakes2_1, group_1, meat2_3, water, coffee1_1, smok_before_0, edema1_0, dried_fruits_1_1, beta_OHB_V1, bread_any1_2, pastries1_1, sweet_drinks1_1, preg_week, fish1_3, AI_V1, alcohol1_1

ah. Predictors: (intercept), carbo, BG0, PG_1h, types_food_n_1, HDLC_V1, climbing_the_stairs1_3, dairy_products2_3, chocolate1_2, whole_grain_bread_1_1, alcohol2_1, coffee2_1, DM_hystory_0, placenta_previa_0, gi, vegetables1_2, cakes2_1, group_1, meat2_3, water, coffee1_1, smok_before_0, edema1_0, dried_fruits_1_1, beta_OHB_V1, bread_any1_2, pastries1_1, sweet_drinks1_1, preg_week, fish1_3, AI_V1, alcohol1_1, fe

ai. Predictors: (intercept), carbo, BG0, PG_1h, types_food_n_1, HDLC_V1, climbing_the_stairs1_3, dairy_products2_3, chocolate1_2, whole_grain_bread_1_1, alcohol2_1, coffee2_1, DM_hystory_0, placenta_previa_0, gi, vegetables1_2, cakes2_1, group_1, meat2_3, water, coffee1_1, smok_before_0,

edema1_0, dried_fruits_1_1, beta_OHB_V1, bread_any1_2, pastries1_1, sweet_drinks1_1, preg_week, fish1_3, AI_V1, alcohol1_1, fe, skimmed_dairy_products1_1

aj. Predictors: (intercept), carbo, BG0, PG_1h, types_food_n_1, HDLC_V1, climbing_the_stairs1_3, dairy_products2_3, chocolate1_2, whole_grain_bread_1_1, alcohol2_1, coffee2_1, DM_hystory_0, placenta_previa_0, gi, vegetables1_2, cakes2_1, group_1, meat2_3, water, coffee1_1, smok_before_0, edema1_0, dried_fruits_1_1, beta_OHB_V1, bread_any1_2, pastries1_1, sweet_drinks1_1, preg_week, fish1_3, AI_V1, alcohol1_1, fe, skimmed_dairy_products1_1, education_1

ak. Predictors: (intercept), carbo, BG0, PG_1h, types_food_n_1, HDLC_V1, climbing_the_stairs1_3, dairy_products2_3, chocolate1_2, whole_grain_bread_1_1, alcohol2_1, coffee2_1, DM_hystory_0, placenta_previa_0, gi, vegetables1_2, cakes2_1, group_1, meat2_3, water, coffee1_1, smok_before_0, edema1_0, dried_fruits_1_1, beta_OHB_V1, bread_any1_2, pastries1_1, sweet_drinks1_1, preg_week, fish1_3, AI_V1, alcohol1_1, fe, skimmed_dairy_products1_1, education_1, bread_any2_2

al. Predictors: (intercept), carbo, BG0, PG_1h, types_food_n_1, HDLC_V1, climbing_the_stairs1_3, dairy_products2_3, chocolate1_2, whole_grain_bread_1_1, alcohol2_1, coffee2_1, DM_hystory_0, placenta_previa_0, gi, vegetables1_2, cakes2_1, group_1, meat2_3, water, coffee1_1, smok_before_0, edema1_0, dried_fruits_1_1, beta_OHB_V1, bread_any1_2, pastries1_1, sweet_drinks1_1, preg_week, fish1_3, AI_V1, alcohol1_1, fe, skimmed_dairy_products1_1, education_1, bread_any2_2, walking2_2

am. Predictors: (intercept), carbo, BG0, PG_1h, types_food_n_1, HDLC_V1, climbing_the_stairs1_3, dairy_products2_3, chocolate1_2, whole_grain_bread_1_1, alcohol2_1, coffee2_1, DM_hystory_0, placenta_previa_0, gi, vegetables1_2, cakes2_1, group_1, meat2_3, water, coffee1_1, smok_before_0, edema1_0, dried_fruits_1_1, beta_OHB_V1, bread_any1_2, pastries1_1, sweet_drinks1_1, preg_week, fish1_3, AI_V1, alcohol1_1, fe, skimmed_dairy_products1_1, education_1, bread_any2_2, walking2_2, vegetables2_raw_3

an. Predictors: (intercept), carbo, BG0, PG_1h, types_food_n_1, HDLC_V1, climbing_the_stairs1_3, dairy_products2_3, chocolate1_2, whole_grain_bread_1_1, alcohol2_1, coffee2_1, DM_hystory_0, placenta_previa_0, gi, vegetables1_2, cakes2_1, group_1, meat2_3, water, coffee1_1, smok_before_0, edema1_0, dried_fruits_1_1, beta_OHB_V1, bread_any1_2, pastries1_1, sweet_drinks1_1, preg_week, fish1_3, AI_V1, alcohol1_1, fe, skimmed_dairy_products1_1, education_1, bread_any2_2, walking2_2, vegetables2_raw_3, coffee1_3

ao. Predictors: (intercept), carbo, BG0, PG_1h, types_food_n_1, HDLC_V1, climbing_the_stairs1_3, dairy_products2_3, chocolate1_2, whole_grain_bread_1_1, alcohol2_1, coffee2_1, DM_hystory_0, placenta_previa_0, gi, vegetables1_2, cakes2_1, group_1, meat2_3, water, coffee1_1, smok_before_0, edema1_0, dried_fruits_1_1, beta_OHB_V1, bread_any1_2, pastries1_1, sweet_drinks1_1, fish1_3, AI_V1, alcohol1_1, fe, skimmed_dairy_products1_1, education_1, bread_any2_2, walking2_2, vegetables2_raw_3, coffee1_3

ap. Predictors: (intercept), carbo, BG0, PG_1h, types_food_n_1, HDLC_V1, climbing_the_stairs1_3, dairy_products2_3, chocolate1_2, whole_grain_bread_1_1, alcohol2_1, coffee2_1, DM_hystory_0, placenta_previa_0, gi, vegetables1_2, cakes2_1, group_1, meat2_3, water, coffee1_1, smok_before_0, edema1_0, dried_fruits_1_1, beta_OHB_V1, bread_any1_2, pastries1_1, sweet_drinks1_1, fish1_3, AI_V1, alcohol1_1, fe, skimmed_dairy_products1_1, education_1, bread_any2_2, walking2_2, vegetables2_raw_3, coffee1_3, prot

aq. Predictors: (intercept), carbo, BG0, PG_1h, types_food_n_1, HDLC_V1, climbing_the_stairs1_3, dairy_products2_3, chocolate1_2, whole_grain_bread_1_1, alcohol2_1, coffee2_1, DM_hystory_0, placenta_previa_0, gi, vegetables1_2, cakes2_1, group_1, meat2_3, water, coffee1_1, smok_before_0, edema1_0, dried_fruits_1_1, beta_OHB_V1, bread_any1_2, pastries1_1, sweet_drinks1_1, fish1_3, AI_V1, alcohol1_1, fe, skimmed_dairy_products1_1, education_1, bread_any2_2, walking2_2, vegetables2_raw_3, coffee1_3, prot, climbing_the_stairs1_1

ar. Predictors: (intercept), carbo, BG0, PG_1h, types_food_n_1, HDLC_V1, climbing_the_stairs1_3, dairy_products2_3, chocolate1_2, whole_grain_bread_1_1, alcohol2_1, coffee2_1, DM_hystory_0, placenta_previa_0, gi, vegetables1_2, cakes2_1, group_1, meat2_3, water, coffee1_1, smok_before_0, edema1_0, dried_fruits_1_1, beta_OHB_V1, bread_any1_2, pastries1_1, sweet_drinks1_1, fish1_3, AI_V1,

alcohol1_1, fe, skimmed_dairy_products1_1, education_1, bread_any2_2, walking2_2, vegetables2_raw_3, coffee1_3, prot, climbing_the_stairs1_1, FR_V1

as. Predictors: (intercept), carbo, BG0, PG_1h, types_food_n_1, HDLC_V1, climbing_the_stairs1_3, dairy_products2_3, chocolate1_2, whole_grain_bread_1_1, alcohol2_1, coffee2_1, DM_hystory_0, placenta_previa_0, gi, vegetables1_2, cakes2_1, group_1, meat2_3, water, smok_before_0, edema1_0, dried_fruits_1_1, beta_OHB_V1, bread_any1_2, pastries1_1, sweet_drinks1_1, fish1_3, AI_V1, alcohol1_1, fe, skimmed_dairy_products1_1, education_1, bread_any2_2, walking2_2, vegetables2_raw_3, coffee1_3, prot, climbing_the_stairs1_1, FR_V1

at. Predictors: (intercept), carbo, BG0, PG_1h, types_food_n_1, HDLC_V1, climbing_the_stairs1_3, dairy_products2_3, chocolate1_2, whole_grain_bread_1_1, alcohol2_1, coffee2_1, DM_hystory_0, placenta_previa_0, gi, vegetables1_2, cakes2_1, group_1, meat2_3, water, smok_before_0, edema1_0, dried_fruits_1_1, beta_OHB_V1, bread_any1_2, pastries1_1, sweet_drinks1_1, fish1_3, AI_V1, alcohol1_1, fe, skimmed_dairy_products1_1, education_1, bread_any2_2, walking2_2, vegetables2_raw_3, coffee1_3, prot, climbing_the_stairs1_1, FR_V1, dairy_products2_1

au. Predictors: (intercept), carbo, BG0, PG_1h, types_food_n_1, HDLC_V1, climbing_the_stairs1_3, dairy_products2_3, chocolate1_2, whole_grain_bread_1_1, alcohol2_1, coffee2_1, DM_hystory_0, placenta_previa_0, gi, vegetables1_2, cakes2_1, group_1, meat2_3, water, smok_before_0, edema1_0, dried_fruits_1_1, beta_OHB_V1, bread_any1_2, pastries1_1, sweet_drinks1_1, fish1_3, AI_V1, alcohol1_1, fe, skimmed_dairy_products1_1, education_1, bread_any2_2, walking2_2, vegetables2_raw_3, coffee1_3, prot, climbing_the_stairs1_1, FR_V1, dairy_products2_1, dairy_products1_2

av. Predictors: (intercept), carbo, BG0, PG_1h, types_food_n_1, HDLC_V1, climbing_the_stairs1_3, dairy_products2_3, chocolate1_2, whole_grain_bread_1_1, alcohol2_1, coffee2_1, DM_hystory_0, placenta_previa_0, gi, vegetables1_2, cakes2_1, group_1, meat2_3, water, smok_before_0, edema1_0, dried_fruits_1_1, beta_OHB_V1, bread_any1_2, pastries1_1, sweet_drinks1_1, fish1_3, AI_V1, alcohol1_1, fe, skimmed_dairy_products1_1, education_1, bread_any2_2, walking2_2, vegetables2_raw_3, coffee1_3, prot, climbing_the_stairs1_1, FR_V1, dairy_products2_1, dairy_products1_2, sausages2_2

aw. Predictors: (intercept), carbo, BG0, PG_1h, types_food_n_1, HDLC_V1, climbing_the_stairs1_3, dairy_products2_3, chocolate1_2, whole_grain_bread_1_1, alcohol2_1, coffee2_1, DM_hystory_0, placenta_previa_0, gi, vegetables1_2, cakes2_1, group_1, meat2_3, water, smok_before_0, edema1_0, dried_fruits_1_1, beta_OHB_V1, bread_any1_2, pastries1_1, sweet_drinks1_1, fish1_3, AI_V1, alcohol1_1, fe, skimmed_dairy_products1_1, education_1, bread_any2_2, walking2_2, vegetables2_raw_3, coffee1_3, prot, climbing_the_stairs1_1, FR_V1, dairy_products2_1, dairy_products1_2, sausages2_2, Legumes1_3

ax. Predictors: (intercept), carbo, BG0, PG_1h, types_food_n_1, HDLC_V1, climbing_the_stairs1_3, dairy_products2_3, chocolate1_2, whole_grain_bread_1_1, alcohol2_1, coffee2_1, DM_hystory_0, placenta_previa_0, gi, vegetables1_2, cakes2_1, group_1, meat2_3, water, smok_before_0, edema1_0, dried_fruits_1_1, beta_OHB_V1, bread_any1_2, pastries1_1, sweet_drinks1_1, fish1_3, AI_V1, alcohol1_1, fe, skimmed_dairy_products1_1, education_1, bread_any2_2, walking2_2, vegetables2_raw_3, coffee1_3, prot, climbing_the_stairs1_1, FR_V1, dairy_products2_1, dairy_products1_2, sausages2_2, Legumes1_3, vegetables1_raw_2

S5. Resulting formulas for PPGR predictive models

The formulas for resulting models in Table 6 (with no polynomial features):

iAUC120 = 1.728 + BG0*-2.557e^{-1} + carbo*9.060e^{-3}

BGRise = 2.335 + BG0*-2.591e^{-1} + carbo*1.125e^{-2} + kcal*-3.244e^{-4} + starch*5.902e^{-3}

BG60 = 2.866 + BG0*6.485e^{-1} + carbo*8.493e^{-3} + starch*6.215e^{-3} + HDLC_V1*-2.091e^{-1}

BGMax = 2.668 + BG0*7.097e^{-1} + gi*4.461e^{-3} + gl*1.737e^{-2} + HDLC_V1*-1.606e^{-1} (with GI/GL)

BGMax = 2.366 + BG0*7.034e^{-1} + N_abortions*1.880e^{-1} + carbo*1.340e^{-2} (without GI/GL)

AUC120 = 0.9634 + BG0*6.931e^{-1} + HbA1C_V1*1.993e^{-1} + N_pregnancies*1.940e^{-2} + b1*-4.170e^{-3} + carbo*7.927e^{-3} + gi*3.665e^{-3} + starch*1.947e^{-3} + BMI*1.230e^{-2} + HDLC_V1*-8.435e^{-2} + HDLC_V1*-3.321e^{-2} + leptin_V1*-1.995e^{-3} (with GI/GL)

AUC120 = 2.090 + BG0*7.288e^{-1} + carbo*9.020e^{-3} + HDLC_V1*-1.375e^{-1} (without GI/GL)

iAUC60 = 8.728e^{-1} + BG0*-1.141e^{-1} + carbo*6.963e^{-3} + gi*2.463e^{-3} + kcal*-2.556e^{-4} + water*3.401e^{-4} (with GI/GL)

iAUC60 = 9.742e^{-1} + BG0*-1.130e^{-1} + carbo*7.967e^{-3} + kcal*-3.033e^{-4} + water*2.903e^{-4} (without GI/GL)

AUC60 = 9.899e^{-1} + BG0*8.854e^{-1} + carbo*6.708e^{-3}
